# Targeting TMEM205 mediated drug resistance in ovarian clear cell carcinoma using oncolytic virus

**DOI:** 10.1186/s13048-022-01054-5

**Published:** 2022-12-07

**Authors:** Uksha Saini, Brentley Q. Smith, Kalpana Deepa Priya Dorayappan, Ji Young Yoo, G. Larry Maxwell, Balveen Kaur, Ikuo Konishi, David O’Malley, David E. Cohn, Karuppaiyah Selvendiran

**Affiliations:** 1 Division of Gynecologic Oncology, Department of Obstetrics and Gynecology, Comprehensive Cancer Center, The Ohio State University Wexner Medical Center, Columbus, OH 43210 USA; 2 Department of Neurosurgery, University of Texas, Health Science Center, Houston, USA; 3 Inova Women’s Service Line and the Inova Schar Cancer Institute, Falls Church, VA USA; 4 Division of GYN/ONC, Kyoto University Graduate School of Medicine, Kyoto, Japan

**Keywords:** TMEM205, Oncolytic virus, Exosomes, Clear cell carcinoma, Platinum resistant

## Abstract

**Background:**

Ovarian clear cell carcinoma (OCCC) accounts for approximately 8–10% of epithelial ovarian cancers in the United States. Although it is rare, OCCC usually presents with treatment challenges and the overall prognosis is far worse than high grade serous ovarian cancer HGSOC. The objective of this study was to examine the therapeutic relevance of combining oncolytic virus with cisplatin for ovarian cancer clear cell carcinoma (OCCC).

**Results:**

We identified that TMEM205, a recently discovered transmembrane protein, contributes to chemoresistance in OCCC cells via the exosomal pathway. Mechanistically, TMEM205 undergoes ligand-independent constitutive endocytosis and co-localizes with Rab11 to contribute to the late recycling endosomes in a clathrin-independent manner. Further, we observed that oncolytic virus (oHSV) pretreatment followed by treatment with cisplatin decreases TMEM205 expression and sensitizes cells to cisplatin in a synergistic manner in OCCC cells. TMEM205 interacts with glycoprotein-C of oHSV post-infection; both of these proteins undergo ubiquitination and ultimately get shuttled outside the cell via exosomes. Thus, we demonstrate the mechanotransduction pathway of TMEM205-mediated chemoresistance along with targeting this pathway using oHSV and cisplatin as a powerful therapeutic strategy for OCCC. oHSV combination with cisplatin inhibits OCCC tumor growth in vivo in immunodeficient and immunocompetent mice models.

**Conclusion:**

Our results suggest that the combination of oHSV and cisplatin in immunocompetent as well as immune deficient OCCC tumor bearing mice reduces overall tumor burden as well as metastatic disease thereby providing survival benefit. Additionally, the detection of TMEM205 in exosomal cargo early in OCCC development has potential to be exploited as a biomarker.

**Supplementary Information:**

The online version contains supplementary material available at 10.1186/s13048-022-01054-5.

## Introduction

Ovarian Clear Cell Carcinoma (OCCC) is a distinct and challenging clinical entity. Although it accounts for less than 10% of epithelial ovarian cancers (EOC) in the United States [1–3] OCCC does not respond well to current chemotherapy, has a high rate of recurrence, and the overall survival rate is dismal. Stage-for-stage, OCCC has a poorer prognosis than high grade serous ovarian cancer (HGSOC) [4–7]. One reason for such poor outcomes is resistance to chemotherapy, though the precise mechanism(s) of resistance remains unclear. While platinum-based chemotherapy is standard first-line intervention for EOC, OCCC does not respond well to this regimen. Multiple theories have been suggested, including an increased DNA repair rate and the low proliferation rate of OCCC cells [8, 9]. To improve outcomes it is critical to find ways to bypass chemoresistance. Recently, Annexin A4, ARID1A, ALK, STAT3, mTOR and the over-expression of EGFR have been implicated in OCCC chemo resistance [10–16]. However, the mechanism of these genes in the development of chemoresistance remains unclear, hindering the design of compounds that could selectively target proteins that play a role in the development and regulation of chemoresistance.

TMEM205 is a 189 aa protein predicted to be a four-membrane pass protein. TMEM205 is a novel transmembrane protein, whose structure and function have not been previously elucidated. Shen et.al [17] first showed that TMEM205 may be linked to cisplatin (CP) resistance but the direct role or mechanism by which this occurs has not been uncovered. In a separate report the same group found that it co-localized with Rab8 [18], which is involved in membrane protein trafficking and secretion. To our knowledge, this is the first study aimed at identifying the mechanistic pathway of TMEM205 mediated chemosresistance and exploiting TMEM205 as a therapeutic target.

Oncolytic viruses (oHSV) are designed to replicate in and lyse tumor cells, and also activate anti-tumor immunity [19, 20]. The FDA recently approved an oncolytic HSV-1 (IMLYGIC®) derived virus for metastatic melanoma, and many other viruses are being investigated as novel anti-cancer agents for ovarian cancer in preclinical studies and in patients. Oncolytic viruses (OV) replicate selectively in tumor cells and lead to tumor cell death [21, 22] thereby serving as efficient biological anti-cancer agents. The viral progeny causes secondary infection and virus spreads within the tumor while sparing normal cells. oHSV is an oncolytic virus which has been shown to be effective for the treatment of a variety of cancer types [23–26]. Combining oncolytic virus-therapy with chemotherapy is also known to produce synergistic action via unclear molecular mechanisms [27–30].

The primary goal of this study was to discern possible proteins involved in OCCC chemo-resistance and to define the exact pathways involved. The secondary aim was to exploit the therapeutic potential of TMEM205 in overcoming chemoresistance in OCCC. The overarching hypothesis investigated was that TMEM205 imparts chemoresistance in OCCC via increasing drug efflux by the exosomal pathway and that the combination oHSV and CP treatment overcomes this by exploiting, in part, the gC-TMEM205 interaction in both in vitro and in vivo conditions.

## Results

### Establishing the role of TMEM205 in ovarian clear cell carcinoma (OCCC)

**Fig. 1:**
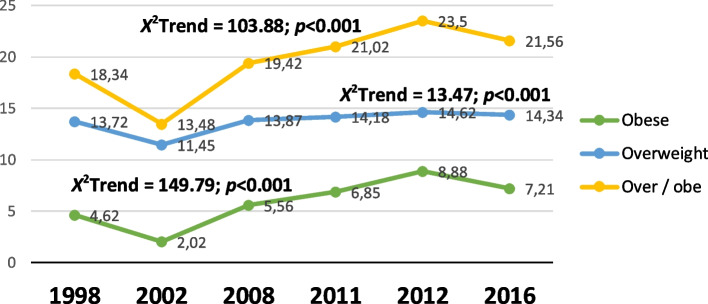
Expression of TMEM205 and its involvement in chemoresistance. **A** SDS PAGE gel stained with Coomassie blue displaying the unique bands in OVTOKO (OV) cells which were sent for protein sequencing. TMEM205 was picked for further evaluation (left panel). Coomassie blue stained SDS PAGE gel for the membrane and nuclear fractions of OVTOKO cells (right panel). **B** WB for the expression of TMEM205 in eight OCCC human samples. **C** Real time quantitative PCR based relative mRNA expression of TMEM205 in 10 OCCC tissues. **D** IHC of tissue from a patient with OCCC showing high expression of TMEM205. **E**) TMEM205 expression showed in OCCC and normal OSE cell lines. **F** OVTOKO cells showing the membrane expression of TMEM205 (green, counterstained with DAPI for nucleus and orange cell mask membrane stain) (**G**) TMEM205 was knocked down in OVTOKO cells. The knockdown was confirmed in two different clones (OV TM Si1 and Si2) using both RT qPCR and WB. We proceeded with OV TM Si1 clone for the further studies. **H** Cell viability SRB assays were observed with scrambled TMEM205 SiRNA (*n* = 5, p0.005). **I** When treated with GFP labeled CP, the OVTOKO cells showed CP localized on the outer membranes of cells (green color) while the OVTOKO TMEM Si cells clearly show CP accumulation in the nuclei (counterstained with red plasma membrane stain)

In an endeavor to identify novel proteins in OCCC, we ran an SDS PAGE gel on total cell lysates extracted from OVTOKO (cisplatin resistant) and A2780 (cisplatin sensitive) cell lines, and stained it with Coomassie blue. We detected two distinct bands in OVTOKO; one high molecular weight band, around 94 kDa, and another low molecular weight band, around 25 kDa (Fig. 1A, right panel). The excised bands were sent for protein identification by MALDI-TOF in which a total of 23 proteins were identified for the lower molecular weight band. The most abundant non-contaminant protein was TMEM205. Since it is known that TMEM205 is a transmembrane protein, we separated the membrane fraction (MF) from the nuclear fraction (NF) of the whole cell lysate for the OVTOKO cells and ran an SDS PAGE gel as well as a WB on both fractions. The 21 kDa band of TMEM205 was quite evident in the MF on the stained gel (Fig. 1A). We then ran a WB on protein lysates of 8 snap frozen human OCCC tissues and observed medium to high expression of TMEM205 (Fig. 1B). Depending on the availability of enough patient tissue (limited quantity for some tissues) for all the samples, we proceeded with extracting RNA from 10 of the human OCCC tissues (including 8 used for the WB) and performed quantitative real-time PCR (q-RT PCR), which displayed medium to high expression of TMEM205 at the RNA level as well (Fig. 1C). Additionally, we performed immunohistochemistry (IHC) on paraffin embedded human OCCC tissues and found very high expression of TMEM205 (Fig. 1D) which was very specific to the primary TMEM205 antibody used (absent in the no primary antibody control). Further, we have observed that TMEM205 expression is significantly elevated in ovarian clear cell carcinoma (OCCC) cell lines, and OCCC tissue than normal ovary epithelial cells (OSE) and ovary benign tissues (Fig. 1E & Sup. Figure 1). At the cellular level, when OVTOKO cells were subjected to ICC, clear membrane specific expression of TMEM205 (green color) was noted in 100% of the cells (Fig. 1F). Having confirmed that the TMEM205 protein is expressed at the cellular as well as the tissue level in OCCC, we proceeded to knock down TMEM205 in OVTOKO cells. Of the 2 clones which showed the least TMEM205 expression at both the RNA and protein level (Fig. 1G), we picked the OVTMSi1 clone to be used in all our future studies. When cell viability was measured using SRB method, we found that the TMEM knockdown (T-siRNA) did not affect the overall cell viability, but when the T-siRNA cells were treated with Cisplatin (T-siRNA+CP), there was a significant 2-fold reduction in viability as compared to any of the controls (Fig. 1H). Given the reports of the association betweenTMEM205 and chemo-resistance, we used GFP labelled cisplatin (10 μM) to treat OVTOKO and OVTMSi cells. The fluorescence images of the treated cells showed that cisplatin accumulated on the periphery of cells in OVTOKO cells but in the TMEMSi cells, all the cells showed cisplatin accumulation within the cell (Fig. 1I). In vitro scratch assays revealed that silencing TMEM205 in OVTOKO cells did not affect cell migration and the wild type (WT) cells, as well as the TMEMSi cells, migrated at the same rates over a 24-hour period (Suppl. Fig. S2). These findings validate the fact that TMEM205 is expressed in OCCC tissues as well as OC cells and may partly be associated with chemo-resistance.

### Mechanotransduction pathways of TMEM205

**Fig. 2:**
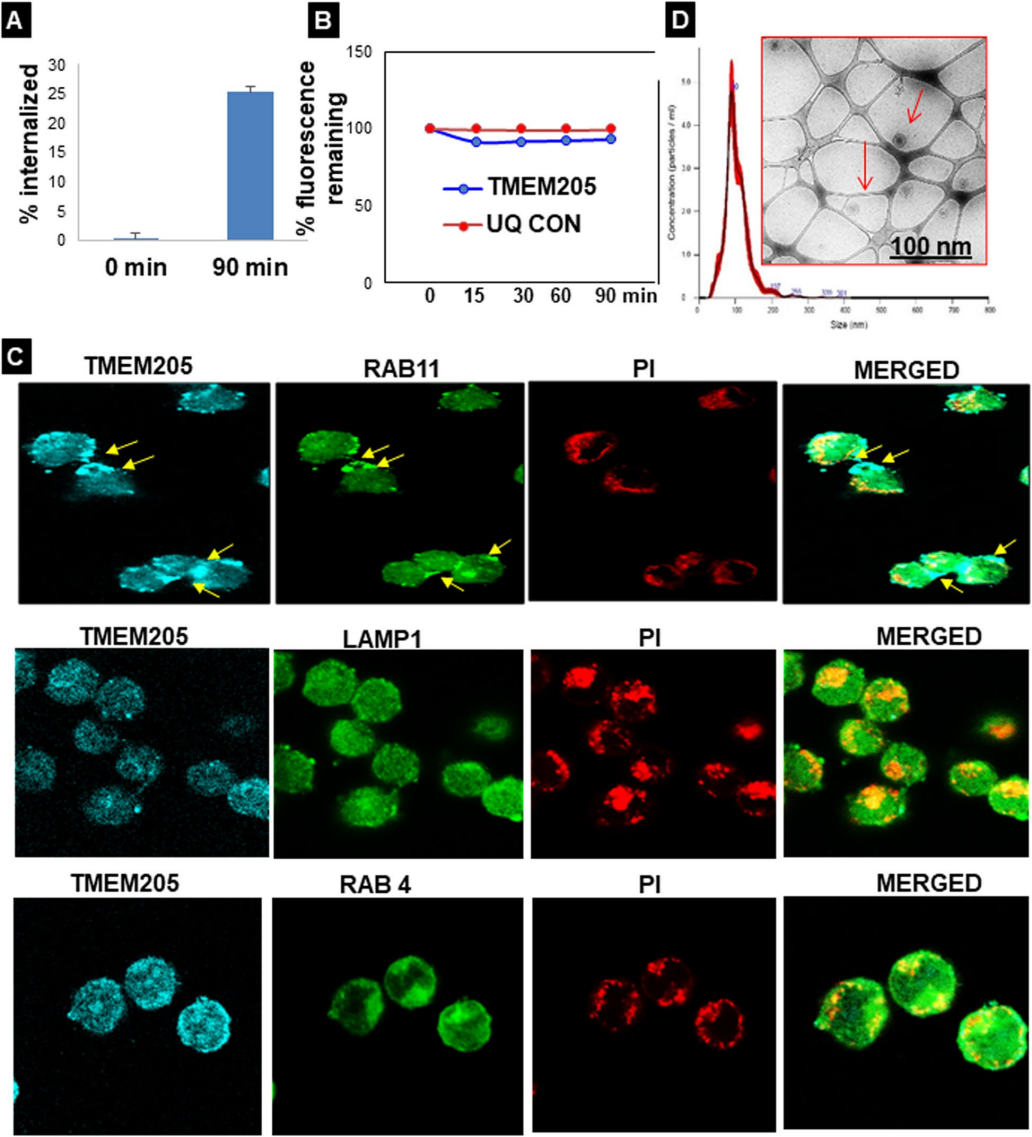
TMEM205 undergoes ligand and clathrin independent endocytic recycling and exosome trafficking. **A** Ab feeding in OVTOKO cells to reveal internalization kinetics of TMEM205. **B** For plasma membrane recycling assay, OVTOKO cells were incubated at 37 °C for 30 min with anti-TMEM205 Ab pre-conjugated with AF488-Fab. The remaining fluorescence from cells at each time point was measured by FCM and plotted as the percentage of pre-recycling samples. **C** Intracellular localization of internalized TMEM205. The internalized TMEM205 was visualized by secondary Ab and examined for co-localization with membrane markers by confocal microscopy for Rab11. Yellow arrowheads indicate vesicles showing co-localization of TMEM205 and Rab11, LAMP1 and RAB4. **D** NTA data of isolated exosomes from OVTOKO cells showing the size and particle concentration distribution plot of exosomes

Since TMEM205 is a newly discovered protein with only five publications to date, knowledge about its physiological role and mechanistic pathways is poor. Thus, we considered it imperative to address the TMEM205 membrane dynamics. We first examined if TMEM205 is constitutively internalized in the absence of ligand using an antibody (Ab) feeding assay combined with a surface fluorescence quenching technique. OVTOKO cells expressing endogenous TMEM205 were labeled for surface TMEM205 with anti-TMEM205 Ab and Alexa Fluor 488 (AF488)-secondary Fab and then incubated at 37 °C for 90 minutes to allow for internalization in the presence of anti-TMEM205 Ab pre-conjugated with AF488-Fab. Remaining surface fluorescence was eliminated with anti-AF488 quenching Ab. The background signal was measured by staining cells with AF488-conjugated isotype IgG2b and analyzed by flow cytometry. The results revealed a steady increase of internalization up to 90 min when ~ 25% of endogenous TMEM205 is internalized constitutively (Fig. 2A). Since the assay was performed in FBS free medium, we conclude that TMEM205 endocytosis is not mediated by the putative FBS-derived ligands.

Many membrane proteins undergo internalization followed by return to the plasma membrane, whereas some are targeted to lysosomes for degradation. To test whether the internalized TMEM205 recycles back to the plasma membrane, we again used an Ab-based surface fluorescence quenching technique. OVTOKO cells were incubated with TMEM205 Ab pre-conjugated with AF488-secondary Fab at 37 °C for 30 min to allow for labeling and endocytosis of TMEM205. After the signal remaining on the cell surface was quenched at 4 °C, the cells were shifted to 37 °C for recycling in the continuous presence of quenching Ab in the medium. Figure 2B shows the time-course change of the remaining fluorescent signal during recycling. The non-quenching Ab control showed only ∼0.52% decrease (red), which might represent signal loss due to receptor degradation. About 0.59% of WT TMEM205 signal was lost in 90 min (blue). Thus, only ∼6% of the endocytosed TMEM205 is recycled to the plasma membrane within 90 min after being internalized; this implies that the remaining TMEM205 either stays in the cytoplasm or is transferred to lysosomes/exosomes for degradation or expulsion. To further investigate the recycling process, we examined the co-localization of internalized TMEM205 with Rab4 and Rab11, markers for the fast and slow recycling endosomes respectively, and the lysosomal marker-LAMP1 using a confocal microscope. TMEM205 co-localized with only Rab11 at 30 min (Fig. 2C. Since Rab11 is located at the intersection between the endocytic and exocytic trafficking pathways, our results suggest that after undergoing internalization, TMEM205 undergoes negligible recycling back to the plasma membrane and is efficiently sorted to the exosome pathway. Having confirmed the association of TMEM205 with Rab11, we further explored the possibility of TMEM205 being present in exosomes secreted by OCCC cells. Exosomes were isolated from FBS free media of OVTOKO cells using Vn96 based ME kit and were confirmed for the right size using Nanoparticle Tracking Analysis (NTA), Image stream Flow cytometry (ISF) and Cryo TEM (Fig. 2D & Sup. Figure 3).

### TMEM205 mediates chemoresistance via the exosomal pathway in OCCC

**Fig. 3:**
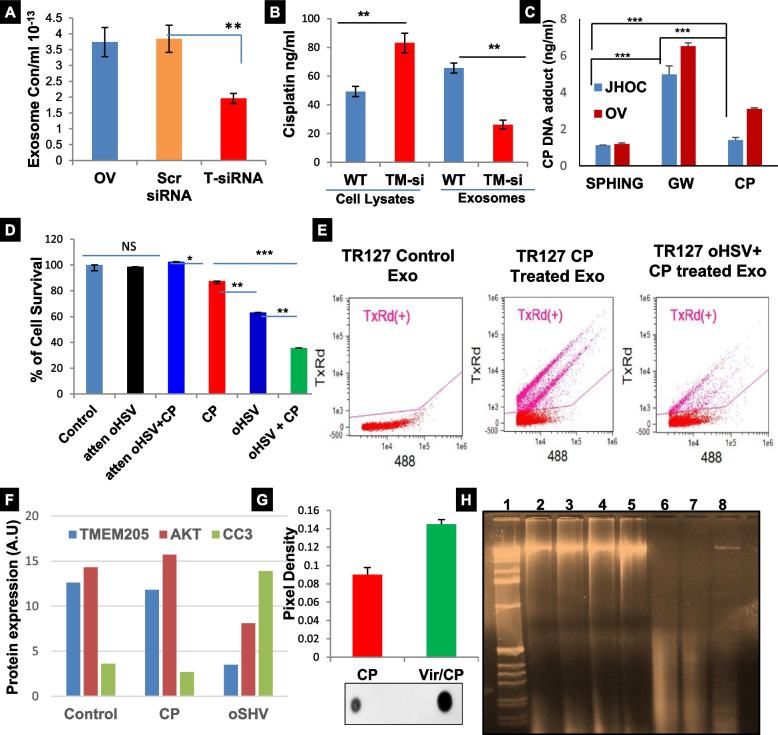
TMEM205 mediates chemoresistance via the exosomal pathway and is overcome by combination oHSV+CP treatment. **A** Exosomes isolated from equal number of OVTOKO cells, OVTOKO cells with TMEM205 knocked down (OV TMSi), or OVTOKO cells with scrambled siRNA (Scr siRNA) control were quantified using NTA (*n* = 4, p0.05). **B** OVTOKO cells and OV TMSi cells were treated with CP; cell-lysates and exosomes were subjected to ICP-MS for analyzing CP accumulation (*n* = 3, p0.001). **C** JHOC and OVTOKO cells were either treated with CP alone or with either GW4869 or sphingomyelinase followed by CP. DNA extracted from the resulting cells was analyzed for CP DNA adduct by ICP MS. **D** SRB assay for OVTOKO cells with CP, oHSV and oHSV+CP. Percent survival was significantly lower in the combination group. Heat killed virus (attenuated oHSV) was used as a control (*n* = 6, p0.05, or 0.01). **E** Flowcytometry of oHSV and CP treated (GFP labeled) or only labeled cisplatin treated OV cells showing red cisplatin. **F** OVTOKO cells were treated with either CP or oHSV alone and the cell lysates were subjected to WB. TMEM205 and Akt were significantly reduced in the cells treated with oHSV. **G** Quantification (pixel density) of dot blot of genomic DNA isolated from OVTOKO cells treated with CP alone or oHSV+CP. H) DNA isolated from cells which were treated with CP or oHSV alone or the combination was run on an 0.8% agarose gel; **H** there was more DNA damage, in the form of shearing, with oHSV treatment (lane 1–8) and in the positive control where the DNA was treated with DNase (Lane1). Here Lane2 is DNA from JHOC cells, Lane 3 is from JHOC cells treated with heat killed oHSV, Lane 4 is from JHOC cells treated with heat killed oHSV followed by CP, Lane 5 is from JHOC cells treated CP, Lane 6 is from JHOC cells treated with oHSV, Lane 7 is from JHOC cells treated oHSV and CP, and Lane 8 is DNA treated with DNase

The confirmation of TMEM205 in exosomes released from conditioned medium led us to the possibility of the involvement of exosomes in chemoresistance. We used the OV TMSi cells and isolated exosomes from the conditioned media as well as from the media of OVTOKO cells and OVTOKO cells transfected with scrambled siRNA. Exosomes were sent for analysis of relative concentrations using NTA and a significantly low concentration of exosomes was present in OVTMSi cells compared to both controls (Fig. 3A). This indicated that higher intracellular TMEM205 expression may be affiliated with increased exosome release. In view of this, we further speculated that the elevated exosome number in the OVTOKO cells might be expelling cisplatin out of the cell, thereby causing cellular chemoresistance. In order to validate this fact, we treated the OVTOKO cells with cisplatin (20 μM) in FBS free media and isolated exosomes. The exosomes as well as the cell lysates were sent for cisplatin detection using Inductively Coupled Plasma-Maas Spec (ICP-MS). The cell lysates from OVTOKO cells had less cisplatin accumulation than the OVTMSi cells and the opposite was seen in relation to the exosome vesicles (Fig. 3B). Hence, it was verified that silencing TMEM205 results in increased intracellular accumulation of cisplatin due to a decrease in the number of exosomes. For additional authentication JHOC and OVTOKO cells were either untreated or treated with sphingomyelinase 1unit/ml [positive control, increases the release of exosomes [31] or GW 4869 20 μM [exosomal release inhibitor [32] for 12 hours, followed by treatment with cisplatin for 24 hours. DNA extracted from the cells was quantified and confirmed by ICP-MS (Fig. 3C). We observed that when exosome release is inhibited using GW4869, there is more cisplatin adduct formed for both OVTOKO and JHOC cells when compared to treatment with cisplatin alone (Fig. 3C). These results suggest that TMEM205 expression plays a key role in the chemoresistance of OC through exosome secretion.

### oHSV- cisplatin combination overcomes chemoresistance in OCCC

Although there is evidence of activation of specific pathways that can be targeted for treatment of OCCC [33, 34], no therapies have proven effective for the treatment of OCCC. Understanding the role TMEM205 plays in chemoresistance drove us to test therapeutic strategies which could target TMEM205 and consequently affect chemoresistance. We performed Sulforhodamine B (SRB) assays to determine a dose response of OVTOKO cells to oHSV alone, cisplatin alone, or the combination of oHSV and CP. Based on these results, we decided to proceed with pre-treatment of oHSV at an MOI of 1 followed by CP treatment at 10 μM. The synergistic effect of oHSV treatment with CP was confirmed using SRB assays with heat-killed oHSV or heat killed oHSV+CP (Fig. 3D Sup. Figure 4A & B) there was no significant difference in cell survival in the latter two cases as compared to the untreated control. To determine if viral infection affected the cellular accumulation of CP, we infected cells with or without oHSV and further treated with CP conjugated to Texas-red. We also performed SRBs with a PI3K inhibitor, an mTOR inhibitor, and sorafenib (a non-specific kinase inhibitor) in combination with CP (Sup. Fig. S5A), but no other treatment was as effective as the oHSV+CP combination. Since most available chemotherapies are ineffective in treating OCCC as single agents there is an interest in identifying novel therapies for this disease that can synergize with existing therapies. Oncolytic viral therapy is one such exciting therapeutic option currently being investigated in OC patients for safety and efficacy (NCT03120624, NCT03225989, NCT02068794 etc). Flow cytometry further revealed significantly increased cisplatin retention in the oHSV+CP combination group in 3 different chemo resistant cell lines (Fig. 3E, right panel; Sup. Fig. S5B). Increased apoptosis was also seen in the oHSV treated cells compared to CP treatment (Sup. Fig. S5C). This indicates that oHSV treatment alone decreases intracellular TMEM205, AKT expression and increased cleaved caspase 3 (Fig. 3F). In an effort to understand the mechanism behind the efficacy of oHSV+CP at the DNA level, we performed dot blots on the total genomic DNA isolated from cells treated with cisplatin alone and the oHSV+CP combination. Dot blots were probed for Anti-Cisplatin modified DNA Ab in order to quantify cisplatin induced DNA adducts. The dot blots from both OVTOKO (Fig. 3G) and JHOC cells (Sup. Fig. S6A) showed significantly more DNA adducts after treatment with the oHSV+CP combination. Similarly, when an agarose gel for total genomic DNA was run with DNA from cells treated with CP, oHSV, heat killed oHSV or oHSV+CP combination, we found that the degradation of DNA was greatest post treatment with the oHSV+CP combination followed by treatment with oHSV alone (Fig. 3H, Sup. Fig. S6B). Western blot of cell lysates from these treatment groups revealed an elevated cleaved PARP and caspase 3 (Sup. Fig. S6C). Collectively, the data shows that the combination treatment of oHSV+CP decreased cell survival while increasing apoptosis and DNA damage via the degradation of TMEM205.

### TMEM205 interacts with glycoprotein C (gC) of oHSV during viral entry and directs it for cellular degradation

**Fig. 4:**
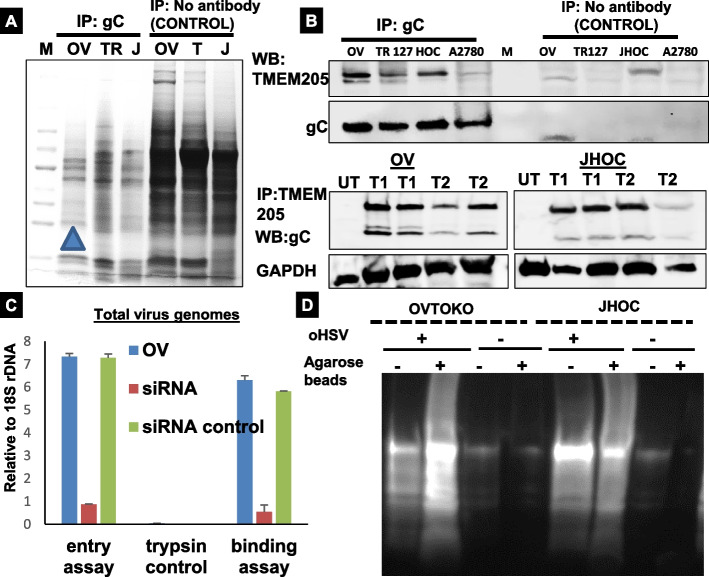
Identification and characterization of TMEM205 as oHSV-interacting host cell protein. **A** Protein complexes directly interacting with oHSV viral particles were isolated by IP using anti-oHSV gC Ab, separated by SDS-PAGE and visualized by Coomassie blue staining. Arrowheads point to some of the proteins differentially identified by mass spectrometry. **B** The WB of the IP samples was probed for human TMEM205. Anti-oHSV gC Ab from oHSV was used for IP. The interaction was further confirmed for OVTOKO and JHOC cells with IP: TMEM205 and WB: gC (lower panel). UT is untreated control; T1 is C-terminal labelled TMEM205 antibody and T2 is N-terminal labelled antibody for both the sets. **C** Wild type OVTOKO cells, TMEM 205 knockdown, and OV TMEM scrambled control cell lines (TM Si) were infected with oHSV to study viral binding and entry. Cells were processed and viral DNA was quantified by qPCR. Data represent the mean ± SEM of three independent experiments. **D** Ubiquitination assay showing ample degradation of TMEM205 mediated by ubiquitin post treatment with oHSV in the first 2 lanes. Thus, oHSV helps in degradation of TMEM205 via the proteasome pathway

On the basis of the results above, we hypothesized that oHSV might be augmenting the degradation of TMEM205 through some novel molecular mechanism(s). To test this hypothesis, we first tried to identify new host cell receptors for oHSV using an immunoprecipitation (IP) approach. OVTOKO, TR127 and JHOC cell proteins were immunoprecipitated using antibodies directed against oHSV glycoprotein C (gC) and glycoprotein D (gD). Precipitated proteins were separated by SDS PAGE gel to select enriched protein bands (Fig. 4A) and the 25kDA band from the OVTOKO cells (Fig. 4A, Lane 2 arrow indicates) was identified by mass spectrometry. Interestingly enough, TMEM205 displayed detectable interaction with gC (Fig. 4B top panel). We further performed WB on these gels and the interaction was confirmed upon IP with gC and WB with TMEM205. In order to specifically identify that which terminal of TMEM205 is involved in the interaction, we used antibody against C or N terminal ends of TMEM205 for IP of OVTOKO and JHOC cells and observed similar pulldown results with gC antibody for both the cases (lanes T1 and T2 in Fig. 4B, lower panel). These results suggested that TMEM205 interacts with gC, which may facilitate binding of host cell TMEM205 to oHSV viral particles.

To further investigate the function of TMEM205 in oHSV binding, viral binding assays (see Materials and Methods) were performed in OVTOKO cells and OVTMSi cells which had a stable knockdown of TMEM205. After incubating for 30 minutes on ice together with oHSV viral particles, we observed up to 6-fold less oHSV binding to OVTOKO cells in the absence of TMEM205 (Fig. 4C). Viral binding to the OVTOKO cell surface without a significant entry was verified by trypsinizing a parallel set of cells at the same time point (Trypsin control in Fig. 4C), which removed up to 96% of the bound virus. Viral entry also decreased in the absence of TMEM205 (Fig. 4C) because of decreased viral binding. These results confirm the significance of TMEM205 in viral binding and subsequent entry into the cells.

Protein ubiquitination is the most important posttranslational modification that controls protein-protein interactions, protein degradation, and spatial localization, which is mediated by chain specific poly-ubiquitination and/or mono-ubiquitination. We utilized this approach to investigate whether oHSV mediated degradation of TMEM205 occurs via the ubiquitin pathway. Western blot analysis of oHSV treated/untreated OVTOKO or JHOC cells with an anti-Ubiquitin Ab reveals a large number of ubiquitination products appearing as a smear or ladder of bands in the presence of oHSV as compared to control lanes. (Fig. 4D). This analysis verifies that the ubiquitination reaction of TMEM205 by oHSV is very efficient. Since we earlier confirmed that TMEM205 mediates chemoresistance through exosomes, we thought it imperative to determine if the gC (oHSV)-TMEM205 axis is tied to exosomes. We first inhibited lysosomal function in OVTOKO cells using ammonium chloride, a selective inhibitor of vacuolar H+ ATPases [inhibits lysosomal movements and phagosome-lysosome (Ph-L) fusion reducing delivery to an intraphagosomally infection in the absence or presence of oHSV (Sup. Fig. S7C). JHOC cells were infected with or without oHSV for 24 hours in the presence or absence of cycloheximide (CHX) or MG132 (proteasome inhibitor). WB was carried out to detect oHSV gC and TMEM205. GAPDH served as loading control (Fig. S7A & B). The protein synthesis inhibitor CHX was used at a concentration of 50 μm. We found that TMEM205 was more stable in the absence of oHSV, whereas the turnover of TMEM205 was more rapid in oHSV treated cells. When exposed to the proteasome inhibitor MG 132 at 20 μm, TMEM205 was protected from degradation. This indicates that TMEM205 is targeted to the proteasome for degradation by oHSV and the absence of a functional proteasome destroys this chemistry.

### In vivo efficacy of oHSV as a single agent or in combination with cisplatin using an orthotopic xenograft tumor model and immunocompetent mice

**Fig. 5:**
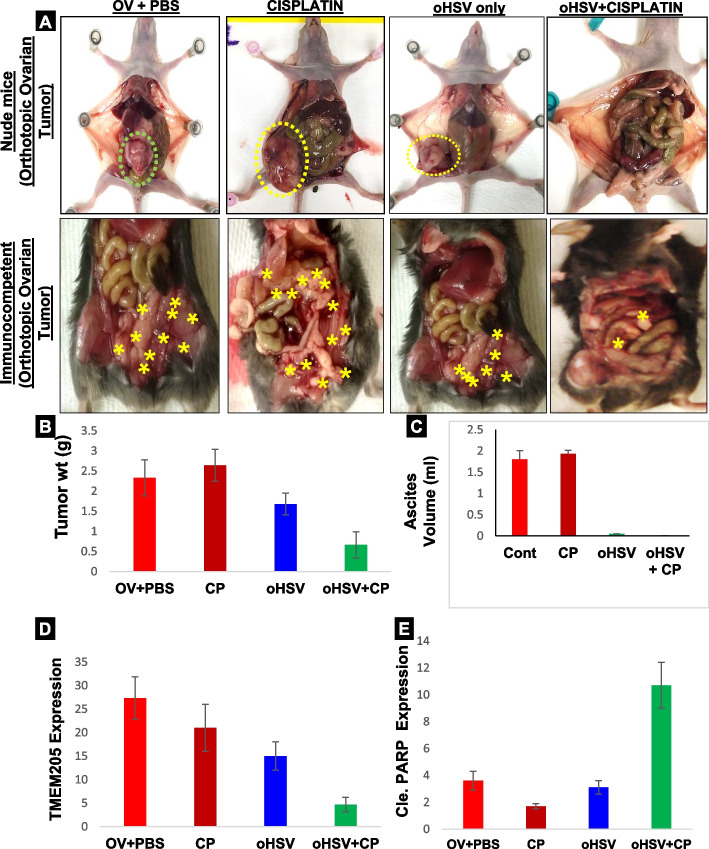
oHSV pre-treatment followed by CP inhibits clear cell cancer proliferation. **A** Immune deficient mice (top panel) were injected with OVTOKO cells orthotopically (in the ovarian bursa) and after 6 weeks were either left untreated (OV + PBS), treated with CP (cisplatin), treated with oHSV (oHSV only) or treated oHSV (week 1) followed by CP (week 2–4, oHSV+CP) treatments were delivered via intraperitoneal injection. Decrease in tumor volume is observed following treatment (*p ≤ 0.05 versus untreated controls.) 6 mice were allocated to each group. Large tumor masses were seen in the ovary and kidney in the untreated mice. For the bottom panel, ID8 cells mixed with mouse derived ascites were injected into the ovaries of immunocompetent mice and the treatment groups were same as for the immune deficient mice. **B** The differences in tumor weight (*n* = 5, *p0.01) and (**C**) ascites volume for 6 immune deficient mice in each group. **D** Western blot of lysates from mice tumor tissues or normal tissues obtained from various groups of treatments. The blot was probed for TMEM205, cleaved PARP, cleaved caspase 3, cleaved caspase 9, and GAPDH. M1 and M2 are different mice from the same group. **E** Tumor-bearing ovaries were excised, and the consecutive tissue sections were stained for rabbit anti-TMEM205 protein Ab to indicate the distribution of TMEM205. High TMEM205 and Ki67 expression is observed in the untreated group and the group treated with CP alone

In order to test whether oHSV will be efficacious when combined with CP in vivo, we used an orthotopic xenograft tumor model in nude mice and an orthotopic tumor model in immunocompetent mice. OVTOKO cells (2 × 10^6^ cells) were injected into the ovarian bursa of immunocompromised mice [] or under the flank skin and the tumor was allowed to develop for 4 weeks. For immunocompetent mice, ID8 cells mixed with murine ascites (1:1; 4 × 10^6^ cells) were injected into the one of the ovarian bursa. Once the tumor was noticeable by MRI the mice were equally divided into groups of 5 for treatment with oHSV alone, cisplatin alone, or oHSV pretreatment (1 dose first week) followed by treatment with cisplatin (2 mg/kg; 1 dose/week for 3 weeks), see schematic figure (Sup. Fig. S8). One group of mice was used as a control and these mice were injected with PBS. Significant overall decrease in tumor volume was observed following the combination treatment versus untreated control (Fig. 5A-C, Sup. Figs. S9 & S10) for all the 3 models across the board. For pathological examination of specimens, all tumor or control samples (ovary and fallopian tube) were probed with anti-TMEM205 for all the treatment groups. Thus, oHSV pretreatment followed by CP works effectively in vitro as well as reducing the tumor burden in vivo and might be an attractive alternative therapy for OC patients. WB of tissues showed decreased TMEM205 and increased cleaved PARP, Caspase 9 and gC in the combination treated group as compared to other treatments (Fig. 5D, E & Sup. Fig. S11). Overall, these results suggest that the efficacy of the oHSV (MOI = 1) plus CP (10 μM) combination on ID8 cells is more effective in downregulating TMEM205 and increasing cleaved caspase 3 compared to single agent therapy.

## Discussion

TMEM205 is a recently discovered protein and its molecular and functional characterization has only begun to emerge. Published data to this point suggests that TMEM205 is a hypothetical membrane protein which may be involved in chemoresistance in epidermoid carcinoma [17] and that Rab8 enhances this chemoresistance [18, 35]. The exact physiological and molecular functionality of TMEM205 is still unknown. The current study is the first to report that in OCCC, i) TMEM205 undergoes clathrin independent constitutive endocytosis but negligible recycling back to the plasma membrane ii) TMEM205 is abundant in OCCC derived exosomes and will be the newest member of the ExoCarta database iii) TMEM205 mediates cisplatin resistance via the exosomal pathway iv) the combination of oncolytic HSV and CP treatment overcomes the TMEM205 mediated chemoresistance in vitro as well as in vivo, and TMEM205 acts as a co-receptor for gC of oHSV, which facilitates trafficking of TMEM205 into exosomes.

The chemo resistant behavior of OCCC carries a poor clinical prognosis due to limited response to conventional therapy and high likelihood of recurrence [36–38]. Novel treatment approaches using new agents without cross-resistance to platinum compounds are being evaluated to treat OCCC. Annexin A4 (AnxA4) has been the most well characterized and studied chemoresistance factor in OCCC and efforts are still ongoing to develop a drug targeting AnxA4 [39]. Because TMEM205 is a transmembrane protein, elucidation of mechanisms that control cell surface expression of TMEM205 will provide an important basis for developing TMEM205-targeting therapeutics. Our quantitative Ab feeding assay in OVTOKO cells revealed constitutive endocytosis of TMEM205 but insignificant recycling back to the plasma membrane, which indicates that the internalized TMEM205 may be targeted to lysosomes for degradation. We further reported the co-localization of internalized TMEM205 with Rab11 but not with Rab4 or LAMP1. Since Rab11 is known to participate in exosome secretion by regulating the interconnection between endocytic, recycling and secretory pathways [40–43], we infer that TMEM205 enters exosomal pathways after ligand independent endocytosis. In line with this and to our surprise, exosomes isolated from serum free media of OVTOKO and JHOC cells did show a high expression of TMEM205supporting that the endocytosed TMEM205 is sorted into endocytic vesicles.

The next question we investigated was if and how TMEM205 mediates chemoresistance via the exosome pathway. TMEM205 knockdown indeed resulted in diminishing exosome number and increased intracellular cisplatin content. With a primary intent to exploit TMEM205 as a therapeutic target, we demonstrated oHSV treatment decreases TMEM205 expression in OVTOKO cells and leads to increased CP accumulation. Cytotoxicity of cisplatin and oncolytic virus-NV1066, via GADD34 upregulation, has been reported in a malignant pleural mesothelioma cell lines [44]. Keeping in mind the fact that viral or bacterial infections can subvert or re-purpose normal cellular processes in the interests of the pathogen, we set out to understand the mechanism and regulations of the key complexes involved in oHSV-TMEM205 synchrony. An unbiased co-IP approach uncovered a direct interaction of TMEM205 with glycoprotein C (gC) of oHSV. Following some of the classical approaches to study viral receptors, we demonstrated that TMEM205 (which is ubiquitously expressed on the host cell surface) interacts with gC to facilitate the internalization of viral particles. Because of the unavailability of separate antibodies for the C-terminal and N-terminal peptide binding sites of TMEM205, it is unclear which end (C or the N terminal) of TMEM205 is involved in the interaction. It is also likely that additional proteins besides TMEM205 are involved in oHSV infection.

Having demonstrated this, we assessed the fate of the TMEM205-gC complex inside the host cell. There was ample ubiquitin mediated cellular degradation of TMEM205 post treatment with oHSV, indicating active involvement of the proteasomal pathway. Previous studies suggested that ubiquitination of both mycobacterial as well as host-derived soluble proteins leads to their trafficking to MVBs and onto exosomes [45, 46]. Our data also indicated that both TMEM205 and gC undergo ubiquitination functions as a tag for delivery to exosomes. This mechanism of packaging and re-release of TMEM205 through exosomes via gC may be responsible for depleting the cells of TMEM205, thereby paving way for an enhanced distribution and cellular response to CP, post oHSV treatment.

The potent therapeutic efficacy of oHSV+CP combination in vitro was replicated in vivo as well for the mouse models that were tested in immunodeficient and immunocompetent mice. The results from our study provide strong rationale for combining oHSV pretreatment with CP in a therapeutic format for OCCC. The fact that both oHSV as well as CP are already FDA approved provides added advantage to clinical utilization of this strategy. Besides being a marker for chemoresistance, the confirmation of TMEM205 in the exosomal cargo demonstrates its potential to be exploited as a fluid-based clinical biomarker. Overall, this study enhances the understanding of the fundamental chemoresistance mechanisms involved in OCCC which mayassist our efforts to develop therapies that are more effective for women with OCCC. Ultimately, we hope this work would lead to a novel clinical application, with TMEM205-targeting oHSV used in combination with standard platinum-based (and other) chemotherapeutic regimens. This would equate to a paradigm shift in how this challenging clinical entity is treated for OCCC.

## Materials & methods

Cell-culture medium (RPMI 1640), fetal bovine serum (FBS), antibiotics, sodium pyruvate, trypsin, and phosphate-buffered saline (PBS) were purchased from Gibco (Grand Island, NY). Polyvinylidene fluoride (PVDF) membrane and molecular-weight markers were obtained from Bio-Rad (Hercules, CA). Antibodies, along with the seller information and dilutions, used in the current study have been listed in Sup. Table 1. Femto Glow Western HRP chemiluminescence reagents were obtained from Michigan Diagnostics (Royal Oak, MI, USA). All other reagents, of analytical grade or higher, were purchased from Cell lines and culture: We have obtained OVTOKO, JHOC and ES2 cell lines from Dr. Ikuo Konishi, Division of Gynecologic oncology, Kyoto, Japan. These cells are very well characterized and published in ovarian clear cell carcinoma research ^1–3^. These are even available for purchase with companies like ExPasy. Cells once thawed were used for only 3 months and we confirmed them for mycoplasma activity using ATCC® Universal Mycoplasma Detection Kit, every 2 months. Once the frozen cells were thawed, they were passaged for 5 times only and discarded thereafter and a fresh vial was thawed.

### Immunocytochemistry (ICC)

Cells in RPMI medium were seeded onto sterile glass coverslips in 6-well plates with an average population of 50,000 cells/well. After 24 hours of culture the cells were washed, fixed, and incubated with primary antibody according to a previously described ICC protocol ^4, 5^. For studies pertaining to Fig. 1 in “Expression of TMEM205 and its involvement in chemoresistance” section, the cells were not permeabilised using Tween-20 and PBS was used in all the steps, to maintain the membrane integrity. For treatment with GFP labeled cisplatin (Michigan Diagnostics), OVTOKO or OV TM Si cells were treated with 10 μM of GFP labeled cisplatin for 6 hours followed by fixing and ICC.

For ICC of internalized TMEM205, OVTOKO cells were plated in 6 well plates with cover slips cells were labeled with anti-TMEM205 Ab for 20 min at 4 °C and then incubated at 37 °C for 15 min to allow for endocytosis To visualize only the internalized TMEM205, the epitope of the TMEM205 Ab on the surface was blocked by incubating the cells with unconjugated goat anti-rabbit IgG at 4 °C. After fixation and permeabilization as described, internalized TMEM205 was labeled with Brilliant Violet conjugated Donkey anti-Rabbit IgG (H + L). Images were collected with FV3000 confocal laser scanning microscope using LSM Image Browser (Zeiss) software. Antibodies used are listed in Table 1. See the detail methods in Supplementary section.

## Supplementary Information

**Additional file 1.**

**Additional file 2.**

## Data Availability

The datasets used and/or analyzed during the current study are available from the corresponding author on reasonable request.

## References

[1] SEER Stat Fact Sheets (2015). Ovary Cancer.

[2] Han L, Zheng A, Wang H (2016). Clear cell carcinoma arising in previous episiotomy scar: a case report and review of the literature. J Ovarian Res.

[3] Siegel RL, Miller KD, Jemal A (2020). Cancer statistics, 2020. CA Cancer J Clin.

[4] Hamanishi J, Mandai M, Ikeda T, Minami M, Kawaguchi A, Murayama T, Kanai M, Mori Y, Matsumoto S, Chikuma S (2015). Safety and antitumor activity of anti-PD-1 antibody, Nivolumab, in patients with platinum-resistant Ovarian Cancer. J Clin Oncol.

[5] Burger RA, Brady MF, Bookman MA, Fleming GF, Monk BJ, Huang H, Mannel RS, Homesley HD, Fowler J, Greer BE (2011). Incorporation of bevacizumab in the primary treatment of ovarian cancer. N Engl J Med.

[6] Chan JK, Teoh D, Hu JM, Shin JY, Osann K, Kapp DS (2008). Do clear cell ovarian carcinomas have poorer prognosis compared to other epithelial cell types? A study of 1411 clear cell ovarian cancers. Gynecol Oncol.

[7] Pectasides D, Pectasides E, Psyrri A, Economopoulos T (2006). Treatment issues in clear cell carcinoma of the ovary: a different entity?. Oncologist.

[8] Itamochi H, Kigawa J, Akeshima R, Sato S, Kamazawa S, Takahashi M, Kanamori Y, Suzuki M, Ohwada M, Terakawa N (2002). Mechanisms of cisplatin resistance in clear cell carcinoma of the ovary. Oncology.

[9] Itamochi H, Kigawa J, Sugiyama T, Kikuchi Y, Suzuki M, Terakawa N (2002). Low proliferation activity may be associated with chemoresistance in clear cell carcinoma of the ovary. Obstet Gynecol.

[10] Mogami T, Yokota N, Asai-Sato M, Yamada R, Koizume S, Sakuma Y, Yoshihara M, Nakamura Y, Takano Y, Hirahara F (2013). Annexin A4 is involved in proliferation, chemo-resistance and migration and invasion in ovarian clear cell adenocarcinoma cells. PLoS One.

[11] Katagiri A, Nakayama K, Rahman MT, Rahman M, Katagiri H, Nakayama N, Ishikawa M, Ishibashi T, Iida K, Kobayashi H (2012). Loss of ARID1A expression is related to shorter progression-free survival and chemoresistance in ovarian clear cell carcinoma. Modern Pathol.

[12] Miyazawa M, Yasuda M, Fujita M, Kajiwara H, Hirabayashi K, Takekoshi S, Hirasawa T, Murakami M, Ogane N, Kiguchi K (2009). Therapeutic strategy targeting the mTOR-HIF-1alpha-VEGF pathway in ovarian clear cell adenocarcinoma. Pathol Int.

[13] Chandler RL, Damrauer JS, Raab JR, Schisler JC, Wilkerson MD, Didion JP, Starmer J, Serber D, Yee D, Xiong J (2015). Coexistent ARID1A-PIK3CA mutations promote ovarian clear-cell tumorigenesis through pro-tumorigenic inflammatory cytokine signalling. Nat Commun.

[14] Matsuzaki S, Yoshino K, Ueda Y, Matsuzaki S, Kakuda M, Okazawa A, Egawa-Takata T, Kobayashi E, Kimura T (2015). Potential targets for ovarian clear cell carcinoma: a review of updates and future perspectives. Cancer Cell Int.

[15] Bixel K, Saini U, Kumar Bid H, Fowler J, Riley M, Wanner R, Deepa Priya Dorayappan K, Rajendran S, Konishi I, Matsumura N (2017). Targeting STAT3 by HO3867 induces apoptosis in ovarian clear cell carcinoma. Int J Cancer.

[16] Yang C, Zhang L, Love-Gregory L, Sun L, Hagemann IS, Cao D (2021). Identification of novel ALK rearrangements in gynecologic clear cell carcinoma. Int J Cancer.

[17] Shen DW, Ma J, Okabe M, Zhang G, Xia D, Gottesman MM (2010). Elevated expression of TMEM205, a hypothetical membrane protein, is associated with cisplatin resistance. J Cell Physiol.

[18] Shen DW, Gottesman MM (2012). RAB8 enhances TMEM205-mediated cisplatin resistance. Pharm Res.

[19] Saha D, Wakimoto H, Rabkin SD (2016). Oncolytic herpes simplex virus interactions with the host immune system. Curr Opin Virol.

[20] Msaouel P, Dispenzieri A, Galanis E (2009). Clinical testing of engineered oncolytic measles virus strains in the treatment of cancer: an overview. Curr Opin Mol Ther.

[21] Hutzen B, Pierson CR, Russell SJ, Galanis E, Raffel C, Studebaker AW (2012). Treatment of medulloblastoma using an oncolytic measles virus encoding the thyroidal sodium iodide symporter shows enhanced efficacy with radioiodine. BMC Cancer.

[22] Iankov ID, Allen C, Federspiel MJ, Myers RM, Peng KW, Ingle JN, Russell SJ, Galanis E (2012). Expression of immunomodulatory neutrophil-activating protein of helicobacter pylori enhances the antitumor activity of oncolytic measles virus. Mol Ther.

[23] Bolyard C, Yoo JY, Wang PY, Saini U, Rath KS, Cripe TP, Zhang J, Selvendiran K, Kaur B (2014). Doxorubicin synergizes with 34.5ENVE to enhance antitumor efficacy against metastatic ovarian cancer. Clin Cancer Res.

[24] Meisen WH, Dubin S, Sizemore ST, Mathsyaraja H, Thies K, Lehman NL, Boyer P, Jaime-Ramirez AC, Elder JB, Powell K (2015). Changes in BAI1 and nestin expression are prognostic indicators for survival and metastases in breast cancer and provide opportunities for dual targeted therapies. Mol Cancer Ther.

[25] Meisen WH, Wohleb ES, Jaime-Ramirez AC, Bolyard C, Yoo JY, Russell L, Hardcastle J, Dubin S, Muili K, Yu J (2015). The impact of macrophage- and microglia-secreted TNFalpha on oncolytic HSV-1 therapy in the glioblastoma tumor microenvironment. Clin Cancer Res.

[26] Yoo JY, Hurwitz BS, Bolyard C, Yu JG, Zhang J, Selvendiran K, Rath KS, He S, Bailey Z, Eaves D (2014). Bortezomib-induced unfolded protein response increases oncolytic HSV-1 replication resulting in synergistic antitumor effects. Clin Cancer Res.

[27] The oncolytic adenovirus DNX-2401 has antitumor activity in glioblastoma. Cancer Discov. 2018;8(4):382. 10.1158/2159-8290.CD-RW2018-031 29475886

[28] Zhang YA, Nemunaitis J, Samuel SK, Chen P, Shen Y, Tong AW (2006). Antitumor activity of an oncolytic adenovirus-delivered oncogene small interfering RNA. Cancer Res.

[29] Garofalo M, Iovine B, Kuryk L, Capasso C, Hirvinen M, Vitale A, Yliperttula M, Bevilacqua MA, Cerullo V (2016). Oncolytic adenovirus loaded with L-carnosine as novel strategy to enhance the antitumor activity. Mol Cancer Ther.

[30] Zeng Y, Li FD, Du JL, Xue YJ, Liu J, Cao X, Wang HZ (2020). Potent antitumor activity of oncolytic adenovirus expressing C/EBPbeta against hepatocellular carcinoma. Apoptosis.

[31] Shamseddine AA, Airola MV, Hannun YA (2015). Roles and regulation of neutral sphingomyelinase-2 in cellular and pathological processes. Adv Biol Regul.

[32] Essandoh K, Yang L, Wang X, Huang W, Qin D, Hao J, Wang Y, Zingarelli B, Peng T, Fan GC (2015). Blockade of exosome generation with GW4869 dampens the sepsis-induced inflammation and cardiac dysfunction. Biochim Biophys Acta.

[33] Bitler BG, Aird KM, Garipov A, Li H, Amatangelo M, Kossenkov AV, Schultz DC, Liu Q, Shih Ie M, Conejo-Garcia JR (2015). Synthetic lethality by targeting EZH2 methyltransferase activity in ARID1A-mutated cancers. Nat Med.

[34] Tan DS, Miller RE, Kaye SB (2013). New perspectives on molecular targeted therapy in ovarian clear cell carcinoma. Br J Cancer.

[35] Rao J, Wu X, Zhou X, Deng R, Ma Y (2020). TMEM205 is an independent prognostic factor and is associated with immune cell infiltrates in hepatocellular carcinoma. Front Genet.

[36] Sugiyama T, Kamura T, Kigawa J, Terakawa N, Kikuchi Y, Kita T, Suzuki M, Sato I, Taguchi K (2000). Clinical characteristics of clear cell carcinoma of the ovary: a distinct histologic type with poor prognosis and resistance to platinum-based chemotherapy. Cancer.

[37] Crotzer DR, Sun CC, Coleman RL, Wolf JK, Levenback CF, Gershenson DM (2007). Lack of effective systemic therapy for recurrent clear cell carcinoma of the ovary. Gynecol Oncol.

[38] Takano M, Tsuda H, Sugiyama T (2012). Clear cell carcinoma of the ovary: is there a role of histology-specific treatment?. J Exp Clin Cancer Res.

[39] Morimoto A, Serada S, Enomoto T, Kim A, Matsuzaki S, Takahashi T, Ueda Y, Yoshino K, Fujita M, Fujimoto M (2014). Annexin A4 induces platinum resistance in a chloride-and calcium-dependent manner. Oncotarget.

[40] Bhuin T, Roy JK (2015). Rab11 in disease progression. Int J Mol Cell Med.

[41] Boulay PL, Mitchell L, Turpin J, Huot-Marchand JE, Lavoie C, Sanguin-Gendreau V, Jones L, Mitra S, Livingstone JM, Campbell S (2016). Rab11-FIP1C is a critical negative regulator in ErbB2-mediated mammary tumor progression. Cancer Res.

[42] D'Agostino L, Nie Y, Goswami S, Tong K, Yu S, Bandyopadhyay S, Flores J, Zhang X, Balasubramanian I, Joseph I (2019). Recycling endosomes in mature epithelia restrain tumorigenic signaling. Cancer Res.

[43] Dong Q, Fu L, Zhao Y, Du Y, Li Q, Qiu X, Wang E (2017). Rab11a promotes proliferation and invasion through regulation of YAP in non-small cell lung cancer. Oncotarget.

[44] Adusumilli PS, Chan MK, Chun YS, Hezel M, Chou TC, Rusch VW, Fong Y (2006). Cisplatin-induced GADD34 upregulation potentiates oncolytic viral therapy in the treatment of malignant pleural mesothelioma. Cancer Biol Ther.

[45] Pluchino S, Smith JA (2019). Explicating exosomes: reclassifying the rising stars of intercellular communication. Cell.

[46] Smith VL, Jackson L, Schorey JS (2015). Ubiquitination as a mechanism to transport soluble mycobacterial and eukaryotic proteins to exosomes. J Immunol.

[47] Okamoto T, Mandai M, Matsumura N, Yamaguchi K, Kondoh H, Amano Y, Baba T, Hamanishi J, Abiko K, Kosaka K (2015). Hepatocyte nuclear factor-1beta (HNF-1beta) promotes glucose uptake and glycolytic activity in ovarian clear cell carcinoma. Mol Carcinog.

