# Correction: Targeting TMEM205 mediated drug resistance in ovarian clear cell carcinoma using oncolytic virus

**DOI:** 10.1186/s13048-023-01111-7

**Published:** 2023-02-13

**Authors:** Uksha Saini, Brentley Q. Smith, Kalpana Deepa Priya Dorayappan, Ji Young Yoo, G. Larry Maxwell, Balveen Kaur, Ikuo Konishi, David O’Malley, David E. Cohn, Karuppaiyah Selvendiran

**Affiliations:** 1 Division of Gynecologic Oncology, Department of Obstetrics and Gynecology, Comprehensive Cancer Center, The Ohio State University Wexner Medical Center, Columbus, OH 43210 USA; 2 Department of Neurosurgery, University of Texas, Health Science Center, Houston, USA; 3 Inova Women’s Service Line and the Inova Schar Cancer Institute, Falls Church, VA USA; 4 Division of GYN/ ONC, Kyoto University Graduate School of Medicine, Kyoto, Japan

**Correction: J Ovarian Res 15, 130 (2022)**

10.1186/s13048-022-01054-5

Following publication of the original article [1], the authors identified an error in Figs. 1, 5 and Additional file. The correct figures are shown below and updated Additional file 2 is also provided in this article. Also, the Ethics approval and consent to participate section was modified, below is the correct statement. The original article has been corrected.

**Fig. 1:**
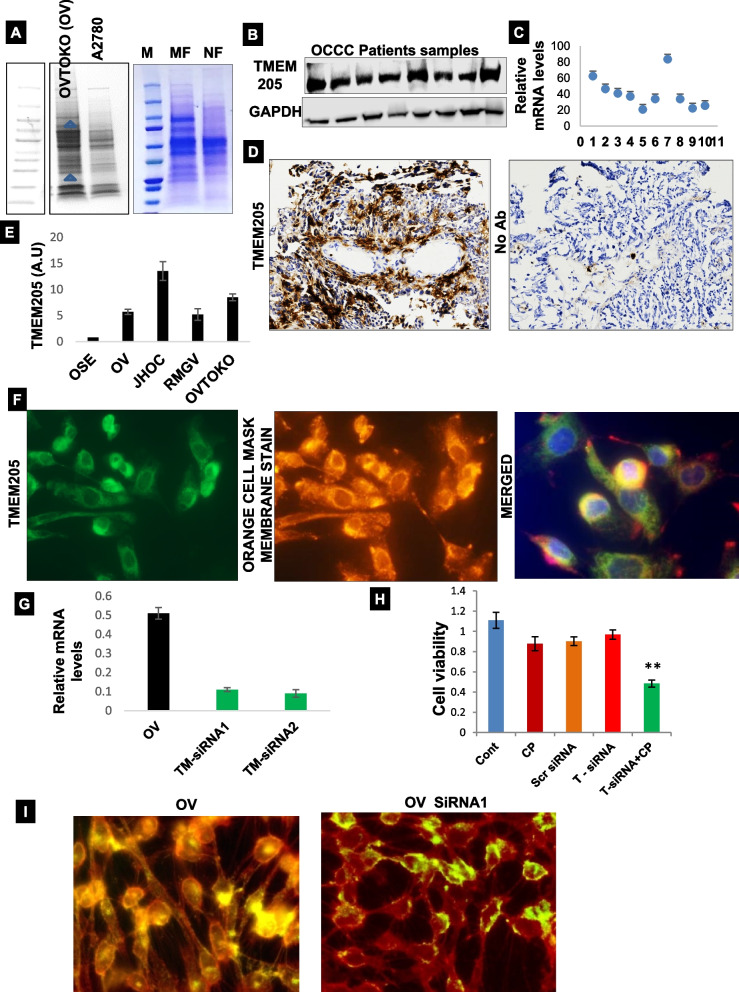
Expression of TMEM205 and its involvement in chemoresistance. **A** SDS PAGE gel stained with Coomassie blue displaying the unique bands in OVTOKO (OV) cells which were sent for protein sequencing. TMEM205 was picked for further evaluation (left panel). Coomassie blue stained SDS PAGE gel for the membrane and nuclear fractions of OVTOKO cells (right panel). **B** WB for the expression of TMEM205 in eight OCCC human samples. **C** Real time quantitative PCR based relative mRNA expression of TMEM205 in 10 OCCC tissues. **D** IHC of tissue from a patient with OCCC showing high expression of TMEM205. **E**) TMEM205 expression showed in OCCC and normal OSE cell lines. **F** OVTOKO cells showing the membrane expression of TMEM205 (green, counterstained with DAPI for nucleus and orange cell mask membrane stain) (**G**) TMEM205 was knocked down in OVTOKO cells. The knockdown was confirmed in two different clones (OV TM Si1 and Si2) using both RT qPCR and WB. We proceeded with OV TM Si1 clone for the further studies. **H** Cell viability SRB assays were observed with scrambled TMEM205 SiRNA (*n* = 5, p0.005). **I** When treated with GFP labeled CP, the OVTOKO cells showed CP localized on the outer membranes of cells (green color) while the OVTOKO TMEM Si cells clearly show CP accumulation in the nuclei (counterstained with red plasma membrane stain)

**Fig. 5:**
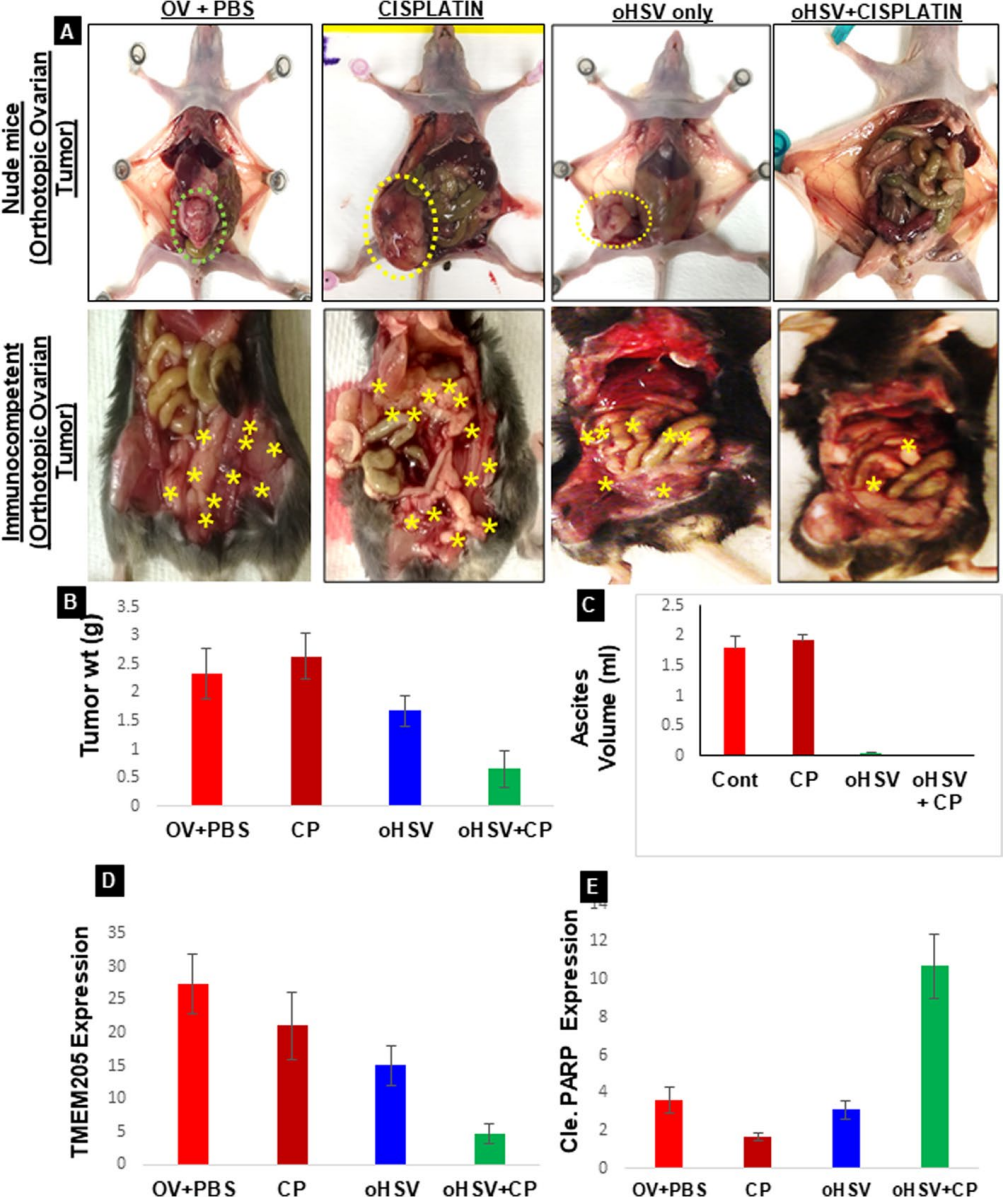
oHSV pre-treatment followed by CP inhibits clear cell cancer proliferation. **A** Immune deficient mice (top panel) were injected with OVTOKO cells orthotopically (in the ovarian bursa) and after 6 weeks were either left untreated (OV + PBS), treated with CP (cisplatin), treated with oHSV (oHSV only) or treated oHSV (week 1) followed by CP (week 2–4, oHSV+CP) treatments were delivered via intraperitoneal injection. Decrease in tumor volume is observed following treatment (*p ≤ 0.05 versus untreated controls.) 6 mice were allocated to each group. Large tumor masses were seen in the ovary and kidney in the untreated mice. For the bottom panel, ID8 cells mixed with mouse derived ascites were injected into the ovaries of immunocompetent mice and the treatment groups were same as for the immune deficient mice. **B** The differences in tumor weight (*n* = 5, *p0.01) and (**C**) ascites volume for 6 immune deficient mice in each group. **D** Western blot of lysates from mice tumor tissues or normal tissues obtained from various groups of treatments. The blot was probed for TMEM205, cleaved PARP, cleaved caspase 3, cleaved caspase 9, and GAPDH. M1 and M2 are different mice from the same group. **E** Tumor-bearing ovaries were excised, and the consecutive tissue sections were stained for rabbit anti-TMEM205 protein Ab to indicate the distribution of TMEM205. High TMEM205 and Ki67 expression is observed in the untreated group and the group treated with CP alone

## Ethics approval and consent to participate

The use of stored human tissues in this study was approved by the Institutional Review Board of the Ohio State University Wexner Medical Center under Study Number: 2004C0124 and the Ohio State University’s OHRP Federal wide Assurance #00006378. No human subjects were directly consented for this study as the tissues were obtained from a biorepository. All procedures used in this study were authorized and conducted according to the guidelines of the Ohio State University Research Institute Ethics Committee. All animal experiments were following the Animal Experimentation Ethics of the Ohio State University Animal Experimentation Research Lab, and the ethics approval number for animal experimentation was 2012A00000008-R3.

## Supplementary Information

**Additional file 2.**
