# Supplementary material for: Targeting TMEM205 mediated drug resistance in ovarian clear cell carcinoma using oncolytic virus
Source: J Ovarian Res. 2022 Dec 7;15:130. doi: 10.1186/s13048-022-01054-5 (PMC9730683; doi:10.1186/s13048-022-01054-5)
Supplement: Supplementary file 1 — Additional file 1. [file 13048_2022_1054_MOESM1_ESM.pdf]

TABLE 1: Antibodies used in the current study

Antibody name	Company (catalogue no.)	Dilution information
TMEM205 (rabbit)	Abcam (ab224516)	WB: 10: 1000 IHC: 2.5:1000 ICC: 10:1000
Anti-HSV1 gC Envelope protein (mouse)	Abcam (ab6509)	WB: 4: 1000
Anti-HSV1 gD (mouse)	Abcam (ab18638)	WB: 4: 1000
EpCam (rabbit)	Cell Signalling Technology (2626S)	WB: 4:1000
B-actin (rabbit)	Cell Signalling Technology (8457)	WB: 1:1000
GAPDH (mouse)	Santa Cruz Biotechnology (SC-32233)	WB: 2:1000
Rab11 (Rabbit)	Abcam (ab3612)	ICC: 4:1000
Rab4 (Rabbit)	Abcam (ab13252)	ICC: 4:1000
Human LAMP1/CD107a Lumenal Domain Biotinylated Antibody (Sheep)	R&D Systems (BAF4800)	ICC: 5:1000
NorthernLights™ 557-conjugated Streptavidin (Sheep)	R&D Systems (NL999)	ICC: 3:1000
Human/Mouse/Rat HSP70/HSPA1A Antibody (Mouse)	R&D Systems (MAP1663-SP)	ICC: 3:1000
Akt (Rabbit)	Cell Signaling Technology (4685)	WB: 2:1000
Cleaved caspase 3	Cell Signaling Technology (9661)	WB: 2:1000
Anti-Cisplatin DNA Adducts Antibody, clone ICR4 (Rat)	Millipore Sigma (MABE416)	Dot Blot: 6:1000
Anti-rat IgG	Millipore Sigma (MAB1663)	Dot Blot: 6:1000
Anti rabbit IgG (HRP)	Abcam(ab6721)	1:1000
Anti mouse IgG (HRP)	Abcam (ab6789)	1:1000
Goat anti-Rabbit IgG (H+L) Highly Cross-Adsorbed Secondary Antibody, Alexa Fluor 488	Invitrogen (A-11034)	2:1000
Goat anti-Mouse IgG (H+L) Highly Cross-Adsorbed Secondary Antibody, Alexa Fluor 568	Invitrogen (A-11031)	2:1000

Alexa fluor 488 Polyclonal antibody	Thermo Fisher Scientific (A-11094)	1:1000
Alexa Fluor® 488 AffiniPure Donkey Anti-Rabbit IgG (H+L)	Jackson Immunoresearch labs. (711-545-152)	10:1000
Rabbit IgG Isotype Control (Alexa Fluor® 488 Conjugate)	Cell signaling technology (4340)	10:1000
AF488-streptavidin	BioLegend (405235)	10:1000

TABLE 2: Reagents/chemicals used in the current study

**Name of compound/kit**	**Vendor (catalogue no.)**	**Concentration used**
Cell mask Orange cell plasma membrane stain	Thermo Fisher Scientific (C10045)	In vitro: 10 µl from the stock in 10 ml RPMI for 2 hours
Cisplatin	Millipore Sigma (PHR1624-200MG)	In vitro: 10µg for 24 hours<br>In vivo: 2mg/kg
GFP-Cisplatin	Michigan Diagnostics	In vitro: 10µg for 5 hours
PYR 41	Millipore Sigma (N2915-5MG**)**	In vitro: 50µM for 2 hours
Dynasore	Millipore Sigma (D7693-5MG)	In vitro: 50µM for 2 hours
Sphingomyelinase	Millipore Sigma (S9396-25UN**)**	In vitro: 1U/10ml for 24 hours
GW4869	Millipore Sigma (D1692-5MG**)**	In vitro: 20µM for 24 hours
Cycloheximide	Millipore Sigma (C7698-1G**)**	In vitro: 50µg/ml for 10 hours
Proteasome Inhibitor 1	**EMD MILLIPORE (539160)**	In vitro: 50µM for 10 hours
MG 132	Millipore Sigma (M8699-1MG**)**	In vitro: 20µM for 10 hours
Lactacystin	Millipore Sigma (L6785-.2MG**)**	In vitro: 10µM for 10 hours
Ammonium Chloride	Millipore Sigma (254134-5G**)**	In vitro: 10mM for 10 hours
Wortmannin (PI3K Inhibitor)	Millipore Sigma (W1628)	In vitro: 1 µM for 24 hours
Torin 1 (mTOR Inhibitor)	Millipore Sigma (475991-10MG)	In vitro: 5 µg for 24 hours
Sorafenib	Reagents Direct (13-D24)	In vitro: 4 µM for 24 hours
DNase I	Millipore Sigma (4716728001**)**	1U/µg of DNA
Propidium Iodide	Millipore Sigma (11348639001**)**	ICC: 1mg/ml for minutes
Pierce Protein A/G Magnetic Agarose Beads	Thermo Fisher Scientific (78609)	IP: 25µl per reaction
Chromatin Condensation and Apoptosis kit	Thermo Fisher Scientific (V23201)	As per manual
The ME™ Kit for Exosome Isolation	New England Peptide (ME-020-kit)	As per manual
DNeasy Blood & Tissue Kit	Qiagen (69504)	As per manual
RNeasy Mini Kit	Qiagen (74104)	As per manual
Transcriptor First Strand Complementary DNA (cDNA) Synthesis Kit	Roche Applied Science (04379012001)	As per manual
iTaq Universal SYBR Green Supermix	Bio-Rad (1725120)	5µl per reaction
PCR Master Mix	Thermo Fisher Scientific (K0171)	5µl per reaction

TABLE 3: Cell lines/primary cells used in the current study

Cell lines name	Origin & Developed place	*Characterization*
OVTOKO	Gifted by our Japanese Collaborators	Ovarian Clear Cell adenocarcinoma, OCCC tumor develop in xenograft, orthotopic and IP mice
JHOC	Ovary, ATCC	Cisplatin Resistant, ARI1DA mutation
RMGV	Ovary, Derived from Japanese patient Stage 4 tumor	Cisplatin Resistant, ARI1DA and PI3K mutation, OC tumor develop in xenograft mice. (Dr. Kunishi lab ref 2015 Clin Can Res)
TR127	Ovary, Developed at Yale University (Dr. Gil Mor)	Cisplatin Resistant, p53 mutation<br>OC tumor growth and metastasis in xenograft mice (Gil Mor MCT 2011)
ID8	Immortalized OC cell lines developed from mouse ovary Gifted from Dr. Ramandeep (UM)	Tumor developed in immunocompetent mice (Ref) Ref. 2017 Science Report
MOCC1	Mouse ovarian cancer ascites derived from the ascites fluid collected from immunocompetent mice injected with ID8 cells. The MOCCs were cultured in medium and mixed with ID8 cells in a 1:1 ratio for injecting back into more immunocompetent mice. The resultant mice develop tumors within 4 weeks of injection in contrary to the 8-10 weeks time taken when injected with ID8 cells only	OC tumor growth and metastasis in orthotopic mice, mimicking the clinical disease observed in patients with ovarian cancer.

MATERIALS AND METHODS

Materials & Methods:

Cell-culture medium (RPMI 1640), fetal bovine serum (FBS), antibiotics, sodium pyruvate, trypsin, and phosphate-buffered saline (PBS) were purchased from Gibco (Grand Island, NY). Polyvinylidene fluoride (PVDF) membrane and molecular-weight markers were obtained from Bio-Rad (Hercules, CA). Antibodies, along with the seller information and dilutions, used in the current study have been listed in **Sup. Table 1**. Femto Glow Western HRP chemiluminescence reagents were obtained from Michigan Diagnostics (Royal Oak, MI, USA). All other reagents, of analytical grade or higher, were purchased from were purchased from Sigma-Aldrich. Cell lines and culture: We have obtained OVTOKO, JHOC and ES2 cell lines from Dr. Ikuo Konishi, Division of Gynecologic oncology, Kyoto, Japan. These cells are very well characterized and published in ovarian clear cell carcinoma research [1-3]. These are even available for purchase with companies like ExPasy. Cells once thawed were used for only 3 months and we confirmed them for mycoplasma activity using ATCC® Universal Mycoplasma Detection Kit, every 2 months. Once the frozen cells were thawed, they were passaged for 5 times only and discarded thereafter and a fresh vial was thawed.

Patient Samples: Patient samples were obtained from biorepositories at both The Ohio State University Wexner Medical Center, Columbus, OH and DR. Maxwell, Division of Gynecologic Oncology Inova Fairfax Hospital, VA. Tissues collected in these biorepositories were from patients undergoing surgery at the respective institutions. In total we received 9 ovarian clear cell carcinoma samples. These were homogenized in non-denaturing lysis buffer and subjected to western blot as per the standard procedure. The use of stored human tissues in this study was approved by the Institutional Review Board (IRB) of The Ohio State University Wexner Medical

Center under Study Number: 2016C0099 and The Ohio State University's OHRP Federalwide Assurance #00006378. No human subjects were directly consented for this study as the tissues were obtained from a biorepository.

Immunocytochemistry (ICC): Cells in RPMI medium were seeded onto sterile glass coverslips in 6-well plates with an average population of 50,000 cells/well. After 24 hours of culture the cells were washed, fixed, and incubated with primary antibody according to a previously described ICC protocol [4, 5]. For studies pertaining to Fig. 1 in "Expression of TMEM205 and its involvement in chemoresistance" section, the cells were not permeabilised using Tween-20 and PBS was used in all the steps, to maintain the membrane integrity.

For treatment with GFP labeled cisplatin (Michigan Diagnostics), OVTOKO or OV TM Si cells were treated with 10 µM of GFP labeled cisplatin for 6 hours followed by fixing and ICC.

For ICC of internalized TMEM205, OVTOKO cells were plated in 6 well plates with cover slips cells were labeled with anti-TMEM205 Ab for 20 min at 4°C and then incubated at 37°C for 15 min to allow for endocytosis To visualize only the internalized TMEM205, the epitope of the TMEM205 Ab on the surface was blocked by incubating the cells with unconjugated goat anti-rabbit IgG at 4°C. After fixation and permeabilization as described, internalized TMEM205 was labeled with Brilliant Violet conjugated Donkey anti-Rabbit IgG (H+L). Images were collected with FV3000 confocal laser scanning microscope using LSM Image Browser (Zeiss) software. Antibodies used are listed in Table1.

Immunoblot analysis: Cell lysates were prepared in non-denaturing lysis buffer as previously described and subjected to immunoblot analyses [6, 7]. For the initial detection experiments, the SDS PAGE gel was stained with coomassie blue. The band appearing around 25kDa was excised and

sent for protein identification to Prot Tech (Phoenixville, PA). Out of 23 proteins identified, the most abundant non contaminant protein was found to be TMEM205 and selected for this study.

Isolation of Membrane Fraction (MF)

We followed the Abcam protocol for subcellular fractionation of membrane proteins. Briefly, OVTOKO cells were lysed in subcellular fractionation buffer (250mM sucrose, 20mM HEPES, 10mM KCl, 1.5 mM MgCl2, 1mM EDTA, 1mM EGTA, 1mM DTT, PI cocktail). , homogenized through a 25G needle for 10 times and incubated on ise for 10 minutes. The pellet was centrifuged at 720G for 5 minutes and centrifuged again in fractionation buffer at 720G for 10 minutes. The pellet is the nuclear fraction (resuspended in standard lysis buffer) while supernatant was centrifuged at 10000G . the supernatant wa centrifuged in an ultracentrifuge at 100,000G for 1hour. The resultant pellet was the membrane fraction which was re-suspended in the standard lysis buffer for the SDS PAGE gel and WB. The WB was probed with anti-TMEM205, EpCAM for confirming MF and with anti β-actin to confirm nuclear fraction.

TMEM205 knockdown: OVTOKO or JHOC cells were transfected using TMEM205 siRNA/shRNA/RNAi Lentivirus (Human) pooled (abm Inc., Richmond BC, Canada, Cat. No. iV024890) as per the manufacturer's protocol. The resulting cells were called as "OVTMSi" cells. Scrambled SiRNA was used as control and resultant transfected cells were called as Scr Si-RNA. The target sequences were:

25 GGCCTGATTAAGATGGTCCATCTACTGGT

127 CGACATACCTTCGGACTAGTGCAGAGCAA

250 TGGGAGGCCAGCCAGCTTTACCTGCTGTT

480 CCATGGGCTGTCCTCTCTTTGCAATCTGG

Changes in TMEM205 target gene expression were measured using qPCR and Western blot along with migration and invasion in comparison to the normal, non-transfected OVTOKO cells.

Cell migration Assay: Cell migration assays were performed on both control OVTOKO and OVTMSi cells using a previously-described wound-healing method [8] and quantified using freehand selection option on Image J software.

Cell viability by SRB assay

Cell viability was determined by a colorimetric assay using Sulforhodamine B (SRB). After the seeded OVTOKO cells (in 96 well plates) achieved ~70% confluency, they were either left untreated or treated with oHSV (MOI-1,2,3,4,5) or ciplsatin (1, 2.5, 5, 10, 20 µM) or; combination of oHSV+virus, with either oHSV constant and CP varying (oHSV=1) or CP constant (10µM) for 6 hours. All experiments were done using six replicates and repeated at least three times as previously described by us[7].

Isolation of exosomes

For isolation of exosomes, OVTOKO or OV TM Si cells with >80% confluence were washed three times with PBS and incubated in FBS free medium for 24 hours. The medium was collected and kept at 4°C until processed and the remaining cells were stored as a pellet at -20°C until further use. Extracellular vesicles were isolated using "The ME™ Kit for Exosome Isolation (Urine/Media)" (New England Peptide Inc., Gardner, MA, USA), following the user's manual. Briefly, the medium was filtered through a 0.2µm syringe filter and 20µl of Vn96 stock was added to the tube and incubated overnight at 4°C with end to end rotation. The tubes were centrifuged at 15,000G for 30 min at RT. After emoving the supernatant, the pellet was redissolved in 1ml of PBS and centrifuged again at 15,000G for 30 min. The resultant

supernatant was discarded and the pellet was either sent for Nanoparticle Tracking Analysis to the OSU Core lab for determination of both the size distribution and relative concentration of microvesicles, as described by us [11]; analysed by Western Blot; or sent to Trace Earth Element Lab at OSU for analyzing the cisplatin content as previously described [11]. Exosomes isolated from pure FBS were used as control exosomes throughout the study.

Treatment of cells with Dynasore or PYR41or Sphingomyelinase or GW 4869 or oHSV

OVTOKO cells were plated in T75 flasks and allowed to attach overnight. Cells were treated for 3 hours with either 80 µM Dynasore (Sigma-Aldrich) or 50mM PYR41 or 1U of sphingomyelinase or 20µM GW4869 or oHSV @ MOI=1. Equivalent volume of the DMSO vehicle was added to control cells. Cells were washed with PBS, and fresh exosome-depleted culture media was added. After 24 h, exosomes were harvested from cell culture supernatant, as described above, and whole-cell lysates were also generated.

Extraction of genomic DNA and DOT BLOT analyses

For dot blot analyses, OVTOKO cells were left untreated or treated with either Sphingomyelinase or GW4869 or Cisplatin or oHSV or OHSV+CP in T75 flasks. Total genomic DNA was extracted using the DNeasy Blood and Tissue kit (Qiagen), following the manufacturer's protocol. The quality and quantity of DNA was measured using Nanodrop. DNA with 260/280 ratio of 1.9 to 2.0 was used for further experiments. 10µg of DNA was sent for estimation of cisplatin-DNA adduct to the Trace Element Lab. Another 10 µg was denatured by boiling at 100°C for 10 minutes and loaded onto a Gene Screen Hybridisation Transfer Nylon membrane. The membrane was crosslinked using a UV Stratalinker for 4 minutes. The membrane was then blocked in 1XTBST and probed with Anti-Cisplatin DNA Adducts Antibody, clone ICR4 (EMD Millipore) in TBST for 18 hours, washed and probed with a rat

secondary HRP antibody for 1 hour, followed by chemiluminescence based detection as described for the WB.

The pixel density of the resulting dots on the membrane was measured using Li-Cor software. Each experiment was repeated 3 times in different sets. The g DNA was also run on a 0.75% agarose gel in 1X TAE and stained with ethidium bromide and pictured using a gel documentation system. OVTOKO control DNA digested with DNAse (1U) was loaded as positive control.

oHSV immunoprecipitation

For oHSV immunoprecipitation experiments, oHSV protein lysates were prepared in PBS without any detergent as previously described [12]. Cells were lysed in PBS containing protease inhibitors using acid washed glass beads (425-600µm, Sigma-Aldrich). oHSV particles @ MOI=1 were incubated with 100 µg of protein lysate for 12 h at 4˚C with constant gentle agitation. oHSV envelope glycoprotein antibodies (gC or gD) were added to the mixture and incubated for another 2 h at 4˚C. Pierce protein A/G magnetic beads (Thermo Fisher Scientific, Cat. No. 88802) were added to the mixture and incubated for another 4 h at 4˚C. Beads were washed 6 times with a wash buffer (20 mM HEPES, 200 mM NaCl, 1 mM EDTA containing 10 mg/ml BSA). One gel was stained with coomassie blue to extract band of interest and another one was blotted on a membrane and probed with anti TMEM205 or gC antibodies.

oHSV binding assay

For the study of oHSV binding, OVTOKO or OV TM Si cells seeded in 6 well plates and incubated with oHSV in duplicates for 30 min on ice. One set was washed with PBS (3X5min) to remove

unbound oHSV particles. The other set of cells were first trypsinized followed by washing with PBS, to remove the bound viral particles. Incubation of cells on ice allows viral binding but prevents viral entry. Total DNA was extracted as explained above and subsequently processed for quantification of viral DNA amount by quantitative PCR (qPCR). oHSV genome equivalents per cells was calculated by determining host cell number by amplification of the PI15 gene as described before [12].

oHSV viral entry assay

For viral entry assay, OVTOKO or OV TM Si cells were incubated with oHSV as mentioned above. The trypsinised as well as non trypsinised cells were subsequently transferred to a 37˚C incubator with fresh media. Both the cell types were grown for another 4 h for viral entry. Total DNA was extracted and used for quantification of viral DNA amount by qPCR [12].

Ubiquitin assay: To trace the ubiquitinated proteins Pierce protein A/G magnetic beads (Thermo Fisher Scientific, Cat. No. 88802) coated with domains having affinity to ubiquitin were incubated in the lysates at 4°C for 2 hours. After washing the beads, the ubiquitinated proteins were subjected to immunoblot for PIAS3 and blotted by the ubiquitin antibody[4].

Treatment with MG132 or proteasome inhibitors

OVTOKO cells were either left untreated (no virus) or treated (+oHSV) with oHSV (MOI=1) and Cycloheximide or MG132 or lactacystin or ammonium chloride or proteasome inhibitor 1 ; as listed in table 2. The cells were collected at 6 time points namely- 0hr, 1hr, 2hr, 4hr, 6hr, 10hr for all the sets. The collected cells were lysed and processed for WB, followed by probing with anti TMEM205 or anti-gC or GAPDH.

In vivo tumor models:

Athymic NCr-nu/nu female mice outbred were provided by the OSUCCC Target Validation Shared Resource (TVSR); the original breeders, Strain number 553 and 554 were purchased either from the NCI Frederick Facility or Charles River. All mice in the study were handled in accordance to the "Guide for the Care and Use of Laboratory Animals" and the "Principles for the Utilization and Care of Vertebrate Animals". All animal experiments were approved by the Institutional Animal Care and Use Committee (IACUC) at the Ohio State University under the Animal use Protocol no. 2012A00000008-R2. All people handling or injecting cells or drugs into mice, removing and measuring tumors, staining and quantifying slides were blinded to the treatment groups. We used 3 mouse models for this study:

a) Xenograft model, where cells (1×10^6 cells in 100 μL of PBS) were injected in the skin beneath the flank (n=6 for each group) of 6-week-old BALB/c nude mice from the OSU Transgenic Mice Core Lab.
b) Intraperitoneal, where cells (1×10^6 cells in 100 μL of PBS) were injected into the peritoneal cavity (n=6 for each group) of 6-week-old BALB/c nude mice from the OSU Transgenic Mice Core Lab.
c) Orthotopic, where cells were implanted into the ovarian bursa (n=6 for each group) (5×10^6 cells in 100 μL of PBS) of 6-week-old BALB/c nude mice from the OSU Transgenic Mice Core Lab as well as the immunocompetent mice.

   The cell lines used are explained in Table 3.

   Additionally, we used 2 kinds of female mice

   - Nude mice obtained from the OSU transgenic Facility

- Immunocompetent mice C57BL/6J (Charles River Labs)

  A mixture of ID8 cells and mouse ascites derived cells (MOCCs) were mixed in a ratio of 1:1. The culture and growth of MADCs is explained in Table 3.

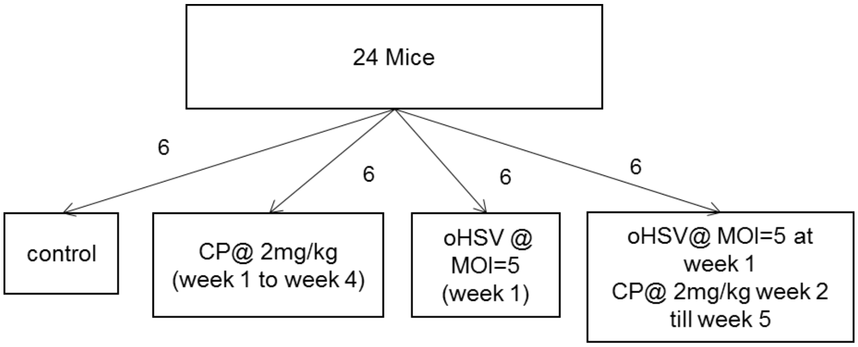

Tumor growth was monitored using MRI and around 2 weeks post injection, the mice bearing similar sized tumors were separated into 4 arms of treatment arms as shown in the adjacent figure. After sacrifice, the tumors were weighed in order to get tumor weight, ascites volume was measured (if present) and metastases sites were counted in the form of number of nodules. The tumor tissues were then subjected to immunoblot analysis and histopathology experiments. Some of the tumor tissues were snap frozen in liquid nitrogen and stored at -80°C for the Real time quantitative PCR.

<u>RNA isolation and Reverse Transcription PCR (RT PCR):</u> Total RNA was isolated from OCCC cells/tissues using the RNeasy Mini Kit (Qiagen, Valencia, CA). RNA samples with an optical density A260/A280 ratio between 1.8 and 2.1 were used. RT-PCR was then performed using the Transcriptor First Strand Complementary DNA (cDNA) Synthesis Kit (Roche Applied Science) to synthesis cDNA. RT-PCR was performed with 1µg of RNA template. The reaction was carried out using the Veriti Thermal Cycler (Applied Biosystems, Carlsbad, CA) and random hexamer primers. The real time quantitative PCR was performed with gene specific primers designed for human GAPDH and TMEM205 (TMEM205: 5′-CTTCCCCTTCTACTTCCACATC-3′ (forward)

and 5′-AGCGTAAGGCTCAGGAAC AG-3′); and SYBR green mix. Each sample was normalized to the control gene glyceraldehyde 3-phosphate dehydrogenase (GAPDH: 5’-AACGGGAAGCTTGTCATCAATGGAAA-3’ and
5’-GCATCAGCAGAGGGGGCAGAG-3’).

.Polymerase chain reaction (PCR)

For PCR, cisplatin-treated DNA samples were used with primers (5′-TATCAGCAATAAACCAGCCA-3′) and (5′-GCGGCCAACTTACTTCTGAC-3′) and PCR Master Mix (Thermo Fisher Scientific) according to the manufacturer’s instructions. PCR products were analyzed by 1 % agarose gel electrophoresis (Sonohara et al., 2015).

Statistical Analysis: Results were expressed as mean ± S.E. Comparisons between groups were made by the Student t-test for all the graphs. The significance level was set at $p \leq 0.05$.
