# Supplementary material for: Targeting TMEM205 mediated drug resistance in ovarian clear cell carcinoma using oncolytic virus
Source: J Ovarian Res. 2022 Dec 7;15:130. doi: 10.1186/s13048-022-01054-5 (PMC9730683; doi:10.1186/s13048-022-01054-5)
Supplement: Supplementary file 2 — Additional file 2. [file 13048_2022_1054_MOESM2_ESM.pdf]

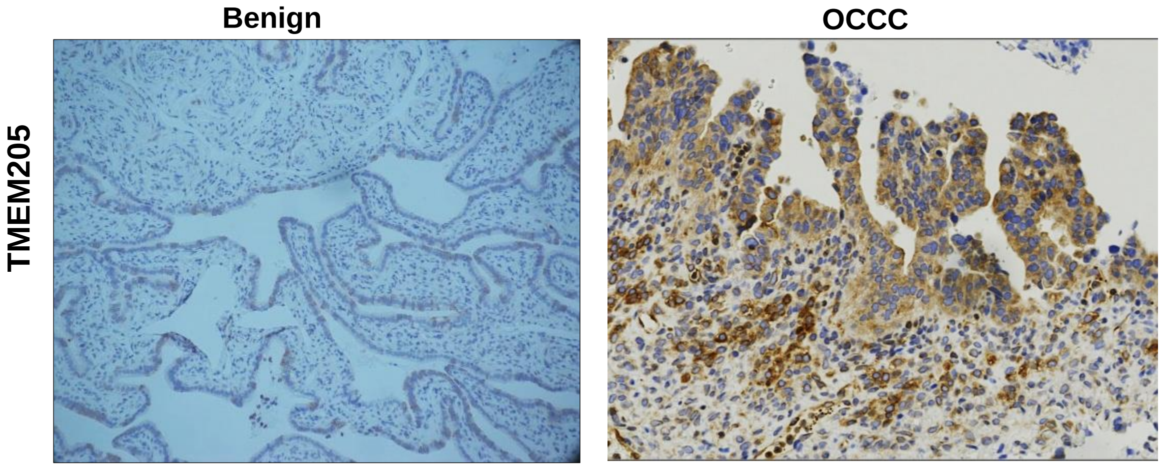

Supplementary Fig. S1: TMEM205 expression is in ovarian benign and clear cell carcinoma (OCCC) tissues.

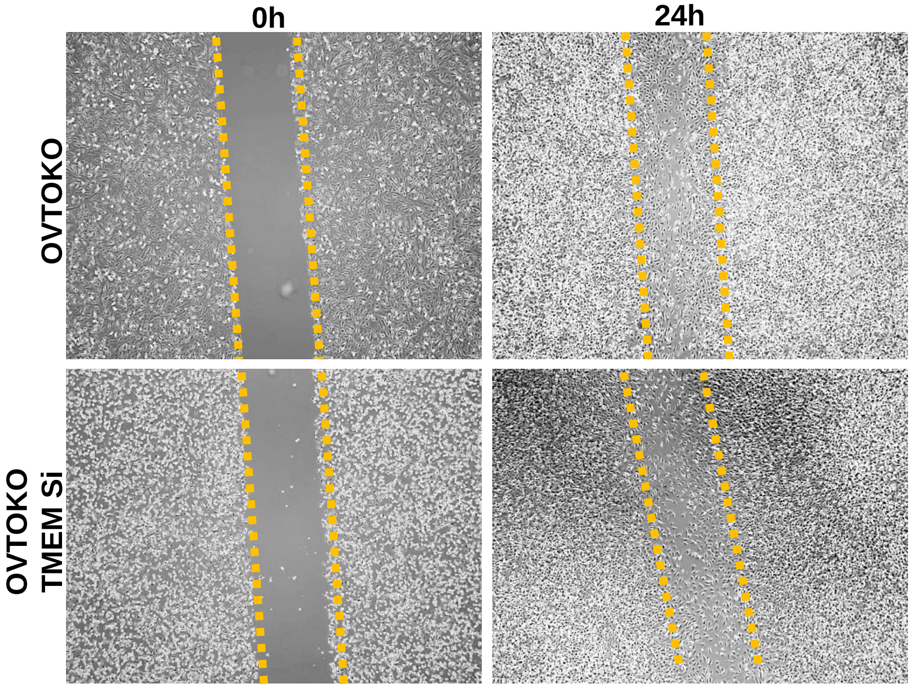

Supplementary Fig. S2: Scratch-wounding cell migration assay. Cells (OVTOKO or OV Tm Si) were plated on 6-well plates (10,000 cells/well) and after letting it grow overnight, a scratch was made with a 1 ml pipette tip in the middle of the well. The cells were allowed to migrate for 24 hours, and migration differences were observed under a light microscope. No difference in the migration capability was noticed in the OVTOKO cells with a knocked down TMEM205.

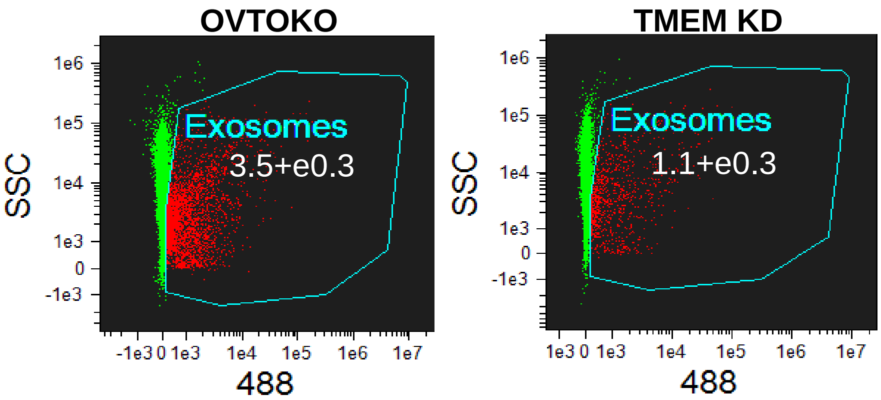

Supplementary Fig. 3 Exosome particles confirmed by flow cytometry (the bar surrounding red color indicates exosome count) in OC cells control and TMEM205 KD cells.

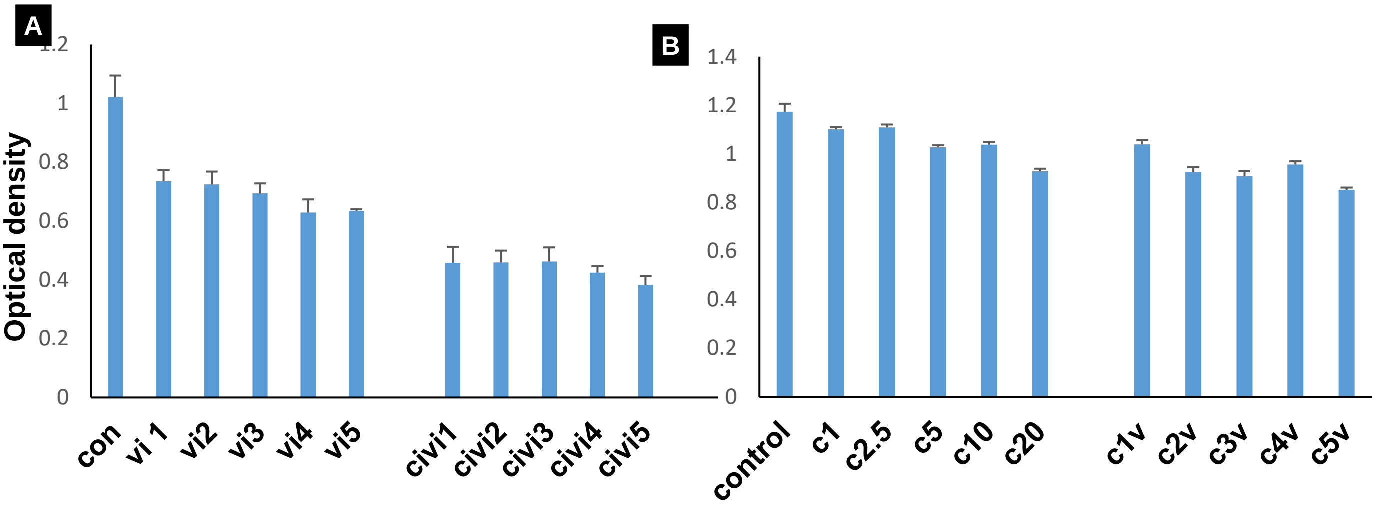

Supplementary Fig. S5A: SRB assays for dose standardization. OVTOKO cells seeded in a 96 well plate (2000 cells/well) were either left untreated or treated with various concentrations of oHSV alone (vi1 to vi5-@MOI 1,2,3,4,5 or oHSV for 1 hour followed by a fixed concentration of cisplatin i.e. 10µM)

Supplementary Fig. S5B: SRB assays for dose standardization. OVTOKO cells seeded in a 96 well plate (2000 cells/well) were either left untreated or treated with various concentrations of cisplatin alone (ci1 to c20-@ 1,2.5,10 and 20µM or with CP and oHSV (MOI=1) for 24 hours

Suppl. Fig. S5

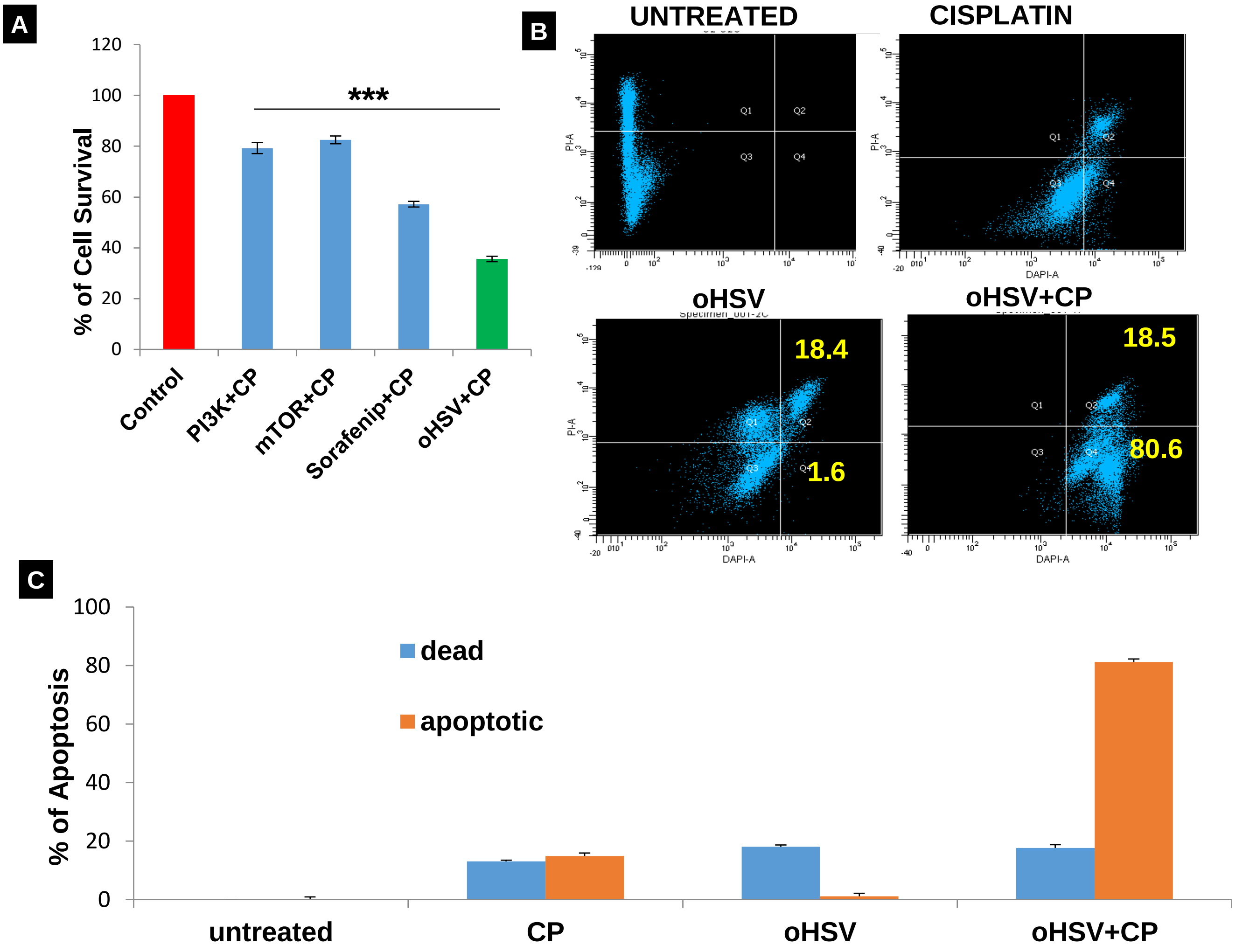

Supplementary Fig. S4A: Cell survival SRB assays with other inhibitors used in clinical trials of OCCC. Cells seeded in a 96 well plate were treated with PI3K inhibitor, mTOR inhibitor, sorafenib or oHSV combined with cisplatin. Supplementary Fig. S4B&C: Percent apoptosis in various groups. OVTOKO cells were either left untreated or treated with CP, oHSV or the combination. The cells, including the floating cells were collected after 24 hours of treatment and analyzed by flow cytometry using chromatin condensation and apoptosis kit.

Suppl. Fig. S6

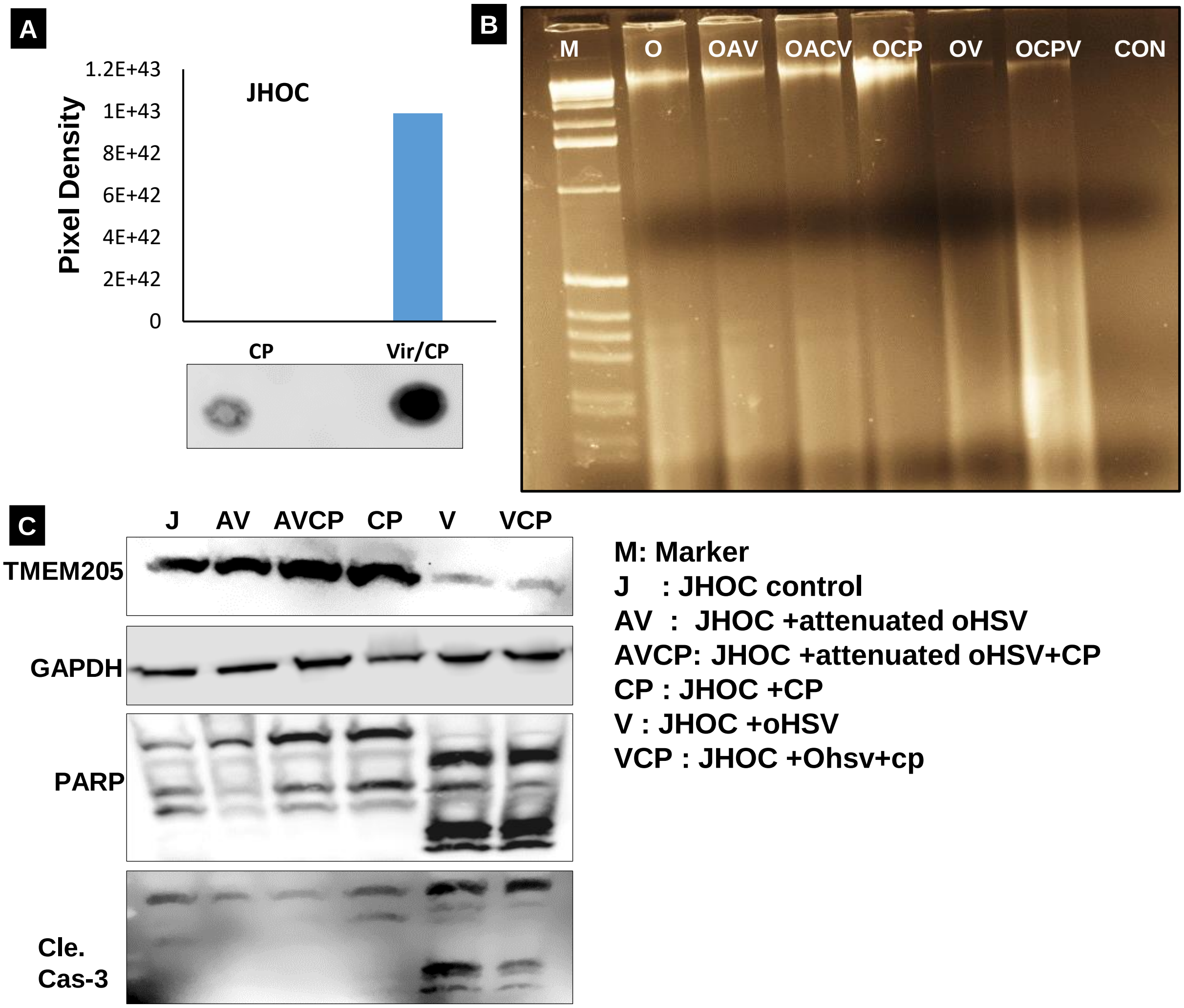

Supplementary Fig. S6A: Dot blot for cisplatin-DNA adduct. JHOC cells were treated with either CP or the combination and DNA from the cells collected after 24 hours was subjected to dot blot. The latter was probed with cisplatin-DNA adduct antibody and the pixel density of the dot was calculated.

Supplementary Fig. S6B: When the genomic DNA isolated from OVTOKO cells treated with CP or oHSV or the comibination was run on a 0.8% agarose gel , there was more DNA damage in the form of shearing in the oHSV treatments (lane OV and OCPV) and in the positive control where the DNA was treated with Dnase (CON). Here O is DNA from OVTOKO cells; OAV is from OVTOKO cells treated with heat killed oHSV; OACV is from OVTOKO cells treated with heat killed oHSV followed by CP; OCP is from OVTOKO cells treated CP; OV is from OVTOKO cells treated with oHSV; OCPV is from OVTOKO cells treated oHSV and CP and Con is DNA treated with DNase

Supplementary Figure S6C: Western blot for apoptosis markers. The protein from cells obtained after treatment as in Supplementary Fig. S10 was subjected to western blot and probed for the difference in the expression of TMEM205 and apoptotic markers cleaved PARP and cleaved caspase3. GAPDH was the loading control.

Suppl. Fig. S7

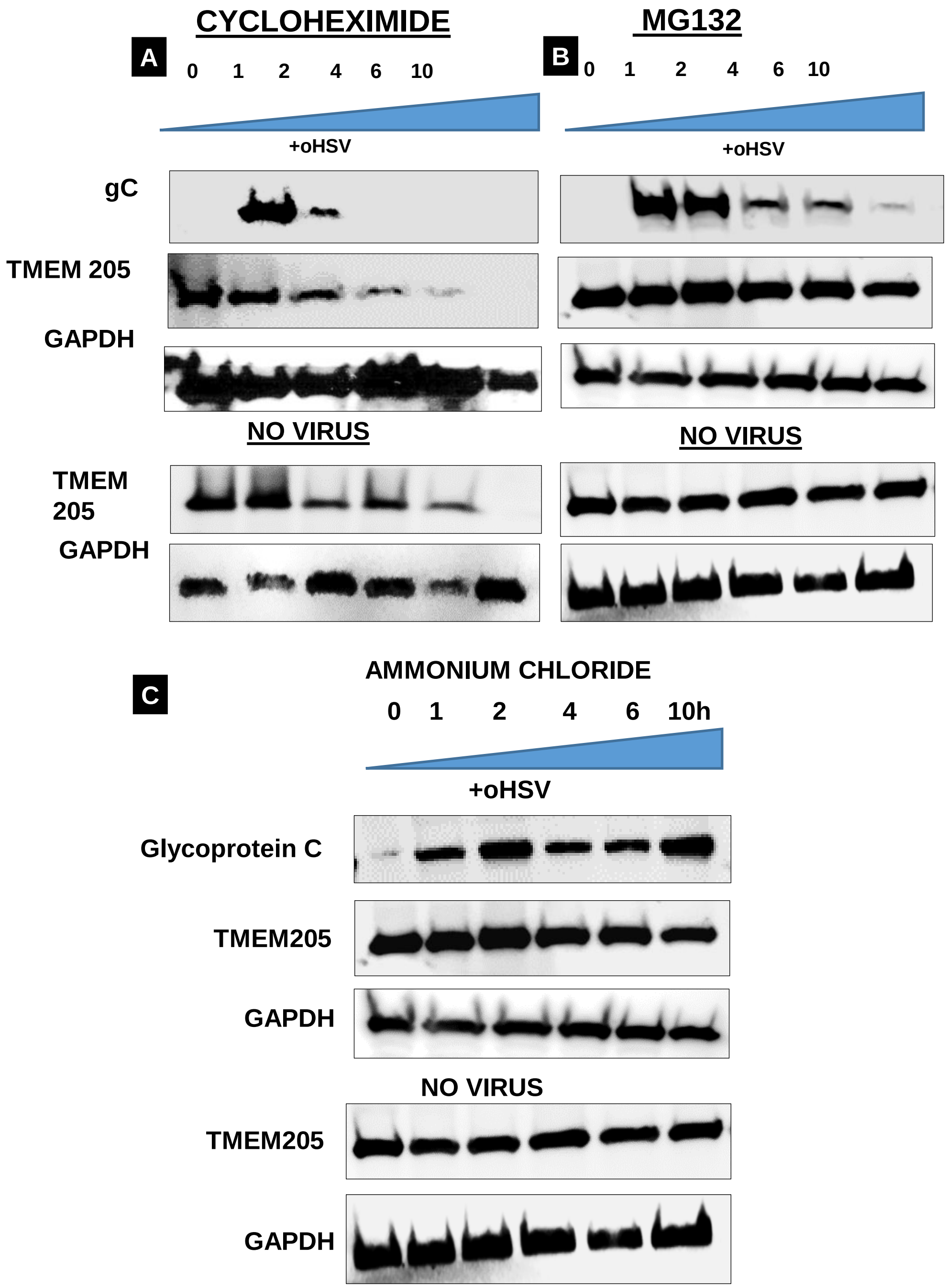

Supplementary Figure S7: **A & B)** JHOC cells were infected with or without oHSV for 24 hours in the presence or absence of cycloheximide (CHX) or MG132 (proteasome inhibitor). WB was carried out to detect oHSV gC and TMEM205. GAPDH served as loading control. The protein synthesis inhibitor CHX was used at a concentration of 50 µm. We found that TMEM205 was more stable in the absence of oHSV, whereas the turnover of TMEM205 was more rapid in oHSV treated cells. When exposed to the proteasome inhibitor MG 132 at 20µm, TMEM205 was protected from degradation. **C**). JHOC cells were infected with or without oHSV for 24 hours in the presence or absence of Ammonium Chloride(lysosome inhibitor), Western blotting was carried out to detect oHSV glycoprotein C and TMEM205. GAPDH served as loading control.

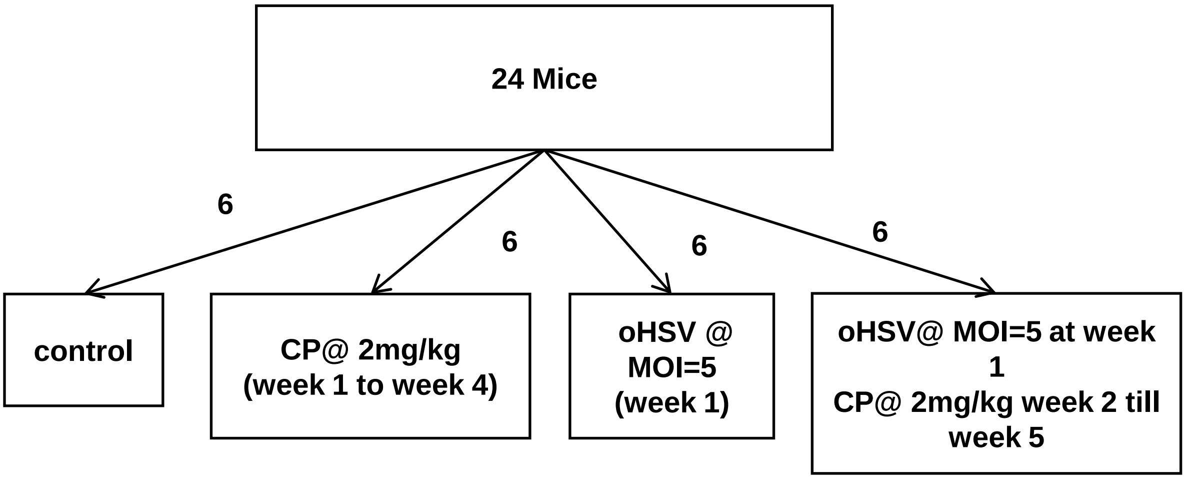

Supplementary Figure S8: Schematic of the mice groups and treatments

Suppl. Fig. S9

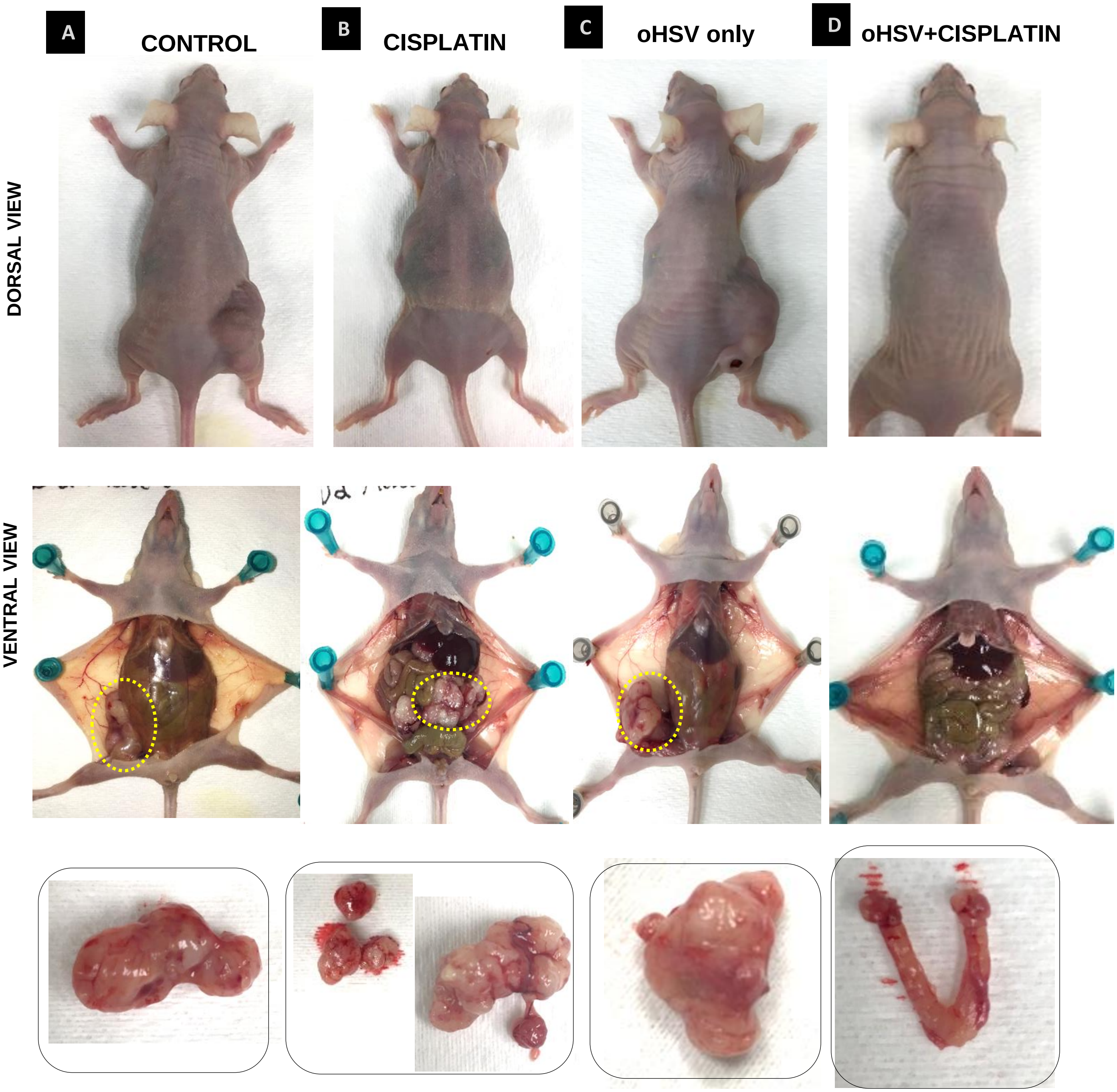

Supplementary Figure S9: oHSV pretreatment followed by cisplatin inhibits tumor growth in nude mice injected with OVTOKO cells IP. OVTOKO **t**umor-bearing mice were left untreated (**A**), treated with cisplatin (**B**) oHSV (**C**) or oHSV followed by cisplatin (**D**) delivered IP for 3 weeks (top panel-dorsal view and middle panel is ventral view). Big tumor masses was seen in the ovary of untreated control and CP treated mice (3rd row). No ovarian tumor in the oHSV +cisplatin treated mice group (bottom graph).

Suppl. Fig. S10

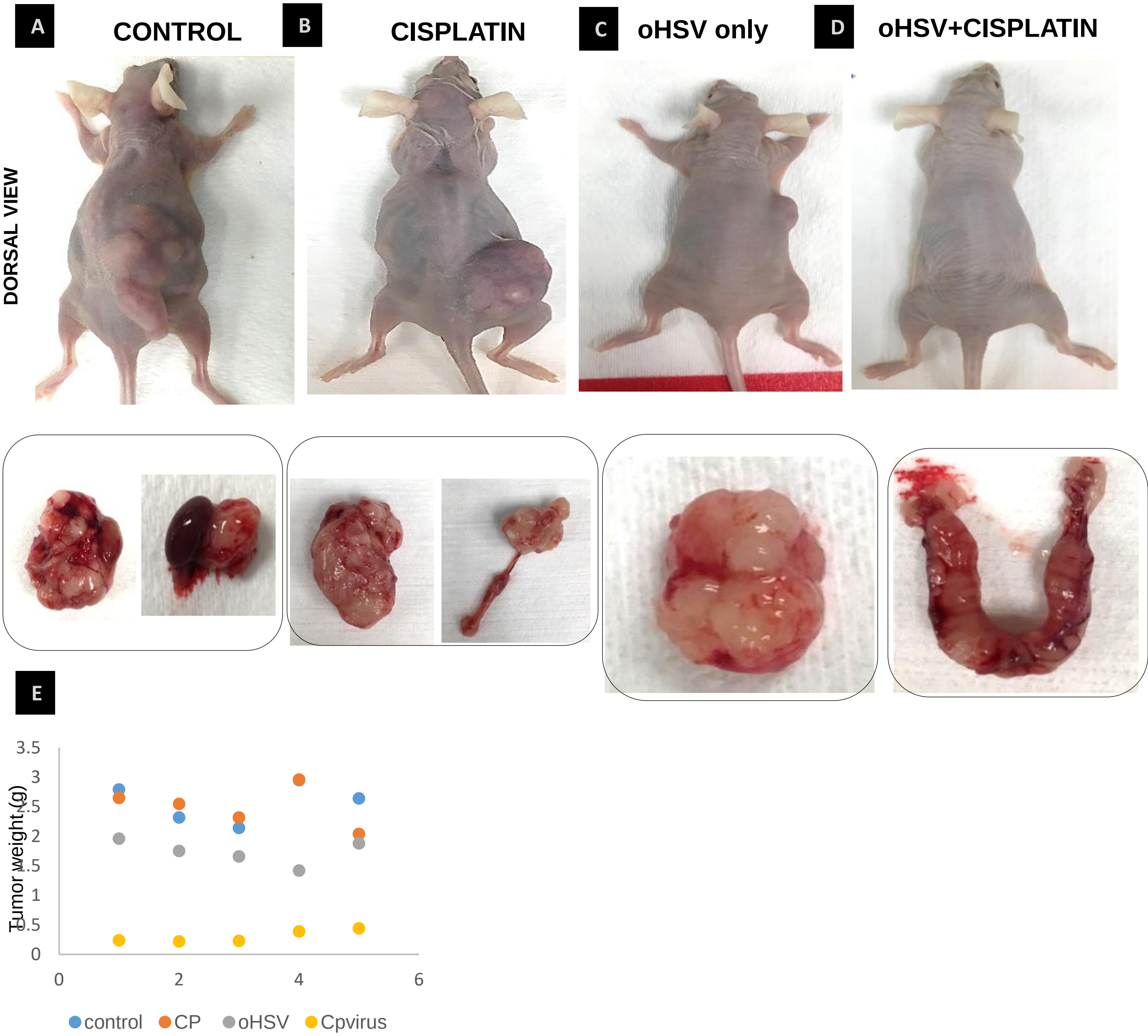

Supplementary Figure S10: oHSV pretreatment followed by cisplatin inhibits tumor growth in nude mice injected with OVTOKO cells orthotopically in the ovary. OVTOKO **t**umor-bearing mice were left untreated (**A**), treated with cisplatin (**B**) oHSV (**C**) or oHSV followed by cisplatin (**D**) delivered IP for 3 weeks (top panel-dorsal view, ventral view in main figure 5). Big tumor masses was seen in the ovary of untreated control and CP treated mice (middle row). (**E**) Tumor weight in the oHSV +cisplatin treated mice group (bottom graph).

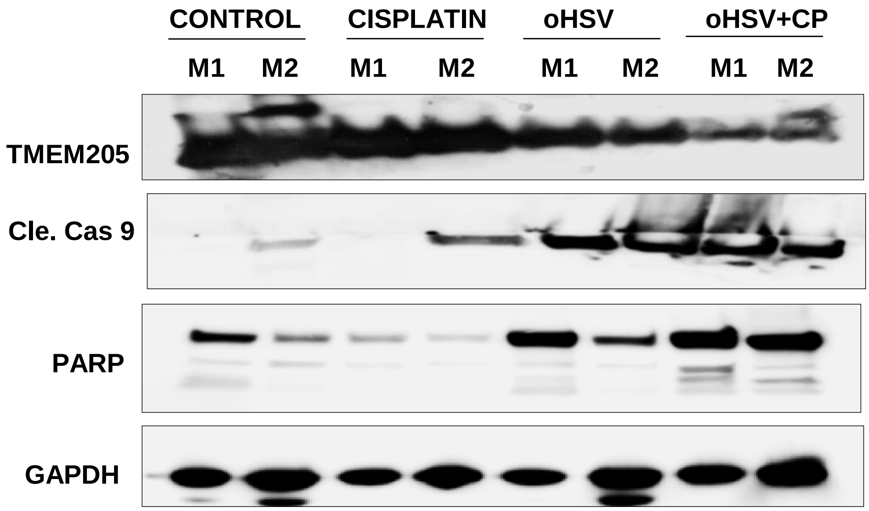

Sup. Fig. S11 Western blot of lysates from mice tumor tissues obtained from various groups of treatments. The blot was probed for TMEM205, cleaved PARP, cleaved caspase 3, cleaved caspase 9, gC and GAPDH. M1 and M2 are different mice from the same group.
